# A Revision of Male Ants of the Malagasy Amblyoponinae (Hymenoptera: Formicidae) with Resurrections of the Genera *Stigmatomma* and *Xymmer*

**DOI:** 10.1371/journal.pone.0033325

**Published:** 2012-03-29

**Authors:** Masashi Yoshimura, Brian L. Fisher

**Affiliations:** Department of Entomology, California Academy of Sciences, San Francisco, California, United States of America; Sheffield University, United States of America

## Abstract

In a male-based revision of ants of the subfamily Amblyoponinae from the Southwest Indian Ocean islands (SWIO: Comoros, Madagascar, Mauritius, Mayotte, Reunion, and Seychelles), we explore and reconsider male morphological characters that distinguish genera within the group. Our investigation redefines *Amblyopone* Erichson sensu Brown (1960), here referred to as *Amblyopone* sensu lato, into three genera: *Xymmer* Santschi **stat. rev.,** *Amblyopone* sensu stricto, *Stigmatomma* Roger **stat. rev.** All species names under *Amblyopone* s. l. reassign into *Xymmer* and *Amblyopone* s. s., which are small, well-defined genera, and *Stigmatomma*, a large group with a generic delimitation that still needs further refinement. Based on a study of male mandible characters and our scenario for mandibular evolution of the worker caste within *Amblyopone* s. l, we conclude that *Amblyopone* s. s. nests outside of XMAS (*Xymmer*+*Mystrium*+*Adetomyrma*+*Stigmatomma*) clade. The following names are transferred from *Amblyopone* s. l. to *Xymmer* as **comb. rev.**: *muticus*. The following names are transferred from *Amblyopone* s. l. to *Stigmatomma* as **comb. rev.**: *amblyops*, *armigerum*, *bellii*, *bierigi*, *bruni*, *celata*, *chilense*, *denticulatum*, *elongatum*, *emeryi*, *feae*, *impressifrons*, *luzonicum*, *minuta*, *normandi*, *oregonense*, *pallipes*, *quadratum*, *reclinatum*, *rothneyi*, *santschii*, *saundersi*, *silvestrii*, *zwaluwenburgi*; as **comb. nov.**: *agostii*, *annae*, *besucheti*, *boltoni*, *caliginosum*, *cleae*, *crenatum*, *degeneratum*, *egregium*, *electrinum*, *eminia*, *exiguum*, *falcatum*, *ferrugineum*, *fulvidum*, *gaetulicum*, *gingivalis*, *glauerti*, *gnoma*, *gracile*, *groehni*, *heraldoi*, *lucidum*, *lurilabes*, *monrosi*, *mystriops*, *noonadan*, *octodentatum*, *ophthalmicum*, *orizabanum*, *papuanum*, *pertinax*, *pluto*, *punctulatum*, *rubiginoum*, *sakaii*, *smithi*, *trigonignathum*, *trilobum*, *wilsoni*, *zaojun*, and *testaceum.* A male-based key to the genera of Malagasy amblyoponine ants, their diagnoses, and a discussion of the evolution of the morphological character of males in the subfamily are given, and the distinguishing characters of each are illustrated. In addition, our results predict that *Paraprionopelta* belongs in the XMAS clade and that *Concoctio* should have males with two mandibular teeth.

## Introduction

Male ants are a largely untapped resource for understanding the taxonomy, phylogeny, diversity, and biology of this important insect group. Although ants are known for having distinctive sexual dimorphism, the current taxonomy of ants is for the most part based on the morphology of female workers. Males are difficult to study because they are often characterized by short emergence periods at certain times of the year, which reduces their chances of capture. Male ant morphology can be equally valuable for identifying species, and among some groups can be even more effective than female traits for distinguishing between genera or species [1]–[5]. The detailed morphological examination of male ants provides new characters that demonstrate phylogenetic relationships [4], [5]. However, morphological information for males is not yet usable for inter-group comparisons due to the lack of comparative studies across taxa. In fact, much of the existing information about male ants is not categorized by taxonomic hierarchy.

In the Malagasy region, we aim to complete a male-based comparative study of the major ant lineages. We have previously published male-based keys to genera and generic diagnoses for Ponerinae [3], Proceratiinae [4], and Dolichoderinae [5] in the Malagasy region. This report adds to the existing body of data by focusing on the subfamily Amblyoponinae.

A few generic keys and synopses for males of Amblyoponinae were found among previous studies. However, no existing key covers the genera in the Malagasy region, and morphological information in those studies was not sufficient to diagnose differences among the Malagasy amblyoponine genera. Emery [6] and Wheeler [7] provide male-based keys to three genera (i.e. *Mystrium, Stigmatomma*, and *Myopopone*), while keys by Kusnezov [8] cover three genera (*Stigmatomma* as *Ericapelta*, *Prionopelta*, and *Paraprionopelta*). Male-based generic synopses have been provided for *Amblyopone* sensu Brown [9] and its related names [6], [9]–[12], for *Myopopone* [6], [9], [10], *Mystrium* [6], [9], [10], *Prionopelta* [9], [10], and *Paraprionopelta* [8]–[10].

**Figure 1:**
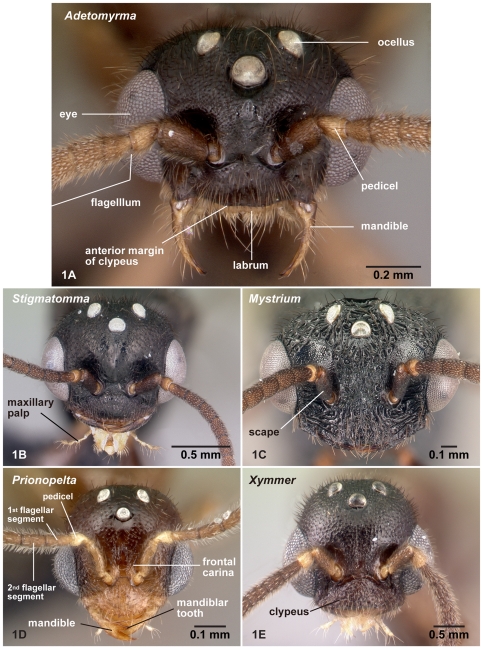
Males of the subfamily Amblyoponinae, head in full-face view. 1A, *Adetomyrma* mg02 (CASENT0079552); 1B, *Stigmatomma* mg01 (CASENT0080397); 1C, *Mystrium* mgm07 (CASENT0081390); 1D, *Prionopelta* mgm05 (CASENT0049679); 1E, *Xymmer* mgm06 (CASENT0083484).

In recent years, molecular phylogenetic analyses have suggested new evolutionary relationships among ant genera and subfamilies, and synergies between molecular and morphological analyses promise to clarify the evolutionary development of ants as a group. Several large-scale studies aiming to clarify the trajectory of global ant evolution [13]–[15] have helped elucidate the relationships among genera in Amblyoponinae. Phylogenetic relationships uncovered via molecular analyses can help identify convergences and plesiomorphies in current taxonomic characters, recast inappropriate groupings based on uninformative characters, and evaluate the utility of morphological characters in each taxonomic rank. For example, figure 1 in the molecular analysis of Brady *et al.* [13] showed that two species of *Amblyopone* sensu Brown [9] included in their study, *Am. pallipes* and *Am. mutica*, belong to different clades, indicating that generic limits need reassessment [16]. Reconsidering the morphological characters of males in Amblyoponinae in light of recent molecular phylogenic results will help evaluate Brown's proposal [17] that male wing characters could be diagnostic for groups within *Amblyopone* sensu Brown [9]. At the same time, a detailed morphological comparative study of male ants will discover new characters supporting molecular phylogenetic hypotheses, helping to clarify intra-subfamilial relationships in Amblyoponinae.

In this study, we provided a key and diagnoses using reconsidered generic characters for five genera in the Malagasy region. Based on the results of a comparative study, we propose resurrecting two names, *Stigmatomma* stat. rev. and *Xymmer* stat. rev., as genera from synonymy within the genus *Amblyopone*. Male synopses for *Adetomyrma* and *Xymmer* are provided for the first time. A questionable concept for the genus *Amblyopone* sensu Brown [9] is discussed on the basis of mandibular characters.

## Materials and Methods

Materials for this work were collected during arthropod surveys in Madagascar and nearby islands in the Southwest Indian Ocean conducted by B. Fisher and Malagasy ant researchers from the Madagascar Biodiversity Center in Antananarivo, Madagascar. Their work in the region includes more than 6,000 leaf litter samples, 4,000 pitfall traps, 1,000 Malaise trap collections, and 9,000 additional hand collection events throughout Madagascar from 1992 through 2009 (see Fisher [18] for additional details). All materials examined other than the types of *Stigmatomma* (*Xymmer*) *muticum* Santschi 1914 (NHMB: see below), *Stigmatomma denticulatum* Roger, 1859 (ZMHB: see below), *Amblyopone australis* Erichson, 1842 (ZMHB: see below), male specimens of *Onychomyrmex* sp., and *Amblyopone australis* (both from ANIC: see below), are at the California Academy of Sciences.

Male specimens were primarily collected with Malaise traps. Within each amblyoponine genus, specimens were sorted to morphospecies. For those species that could not be named, morphospecies codes were applied. The codes consist of a two-letter country code followed by a number, e.g. *Adetomyrma* mg01.

Observations and dissections were carried out under stereoscopic microscopes (LEICA MZ12 and M125). Digital color images were created using a JVC KY-F75 digital camera. Syncroscopy Auto-Montage (v 5.0) software was used for images taken at magnifications less than 100×, and a compound microscope (Leica DM4000M) and Nikon digital camera (DXm1200) Helicon Focus version 4.10.2 software were used for images taken at magnifications greater than 100×. The images were edited in Adobe Photoshop and Illustrator. Each imaged or dissected specimen is uniquely identified with a specimen-level identifier (e.g. CASENT0003099) affixed to each pin.

The male and worker specimens listed below were examined to establish a key to genera of the subfamily. A diagnosis and analysis of morphological evolution is provided for each. Taxon names are followed by a letter code indicating the source of morphological information used to establish the key, and a CASENT specimen identifier of the dissected specimens:

[g]: male specimens that were collected from a colony and associated with workers.

[m]: male specimens that were collected alone and not associated with workers, typically in Malaise traps.

[w]: worker specimen.

[r]: information about male morphologies obtained from published studies. In these cases, the references are shown in brackets.

### Amblyoponinae *Adetomyrma* Ward

*Ad.* mg01 [g: CASENT0218010]; *Ad.* mg02 [g: CASENT0218011]; *Ad.* mg03 [m: CASENT0218012]; *Ad.* mg05 [g: CASENT0218013]; *Ad.* mgm01 [m: CASENT0063101]; *Ad.* mgm02 [m: CASENT0072301]; *Ad.* mgm03 [m: CASENT0111802]; *Ad.* mgm04 [m: CASENT0218014]; *Ad.* mgm05 [m: CASENT0081348].

### *Amblyopone* Erichson

#### Type material

*Amblyopone australis* Erichson, 1842. Lectotype [here designated]. Worker. AUSTRALIA: Tasmania. Scheyer [sic] (misspelling of Schayer) [Museum für Naturkunde der Humboldt-Universität Berlin (ZMHB): 7228, CASENT0104575. Examined based on images. Two specimens were found with same locality and collector information; one was labeled “Type.” The original description of this species does not include detailed type information; however, Erichson mentions in the same paper that Schayer provided much material from Tasmania [19]. Original labels on this specimen seem to have been replaced by new labels, and the collector on the new label is listed as “Scheyer,” a misspelling of Schayer, the collector's name.]

#### Non-Malagasy material

*Am. australis* Erichson, 1842 [w]; *Am. australis* [m: The Australian National Insect Collection (ANIC) 32016150, CASENT0172246. Examined based on images].

### *Apomyrma* Brown, Gotwald & Lévieux

*Ap*. cf01 [m: CASENT0086291, not dissected]; *Ap*. cf02 [m: CASENT0086073, not dissected].

### *Myopopone* Roger

Only genus rank information was available [r: Emery 1911 [6], Brown 1960 [9], and Bolton 2003 [10]]

### *Mystrium* Roger

*M. mysticum* Roger, 1862 [g: CASENT0080864]; *M. oberthueri* Forel, 1897 [g]; *M. rogeri* Forel, 1899 [g: CASENT0218102]; *M.* mg05 [g: CASENT0109124]; *M.* mgm02 [m: CASENT0078803]; *M.* mgm07 [m: CASENT0081493]; *M.* mgm08 [m: CASENT0084212]; *M.* mgm09 [m: CASENT0080644]; *M.* mgm10 [m: CASENT0498897]; *M.* mgm11 [m]; *M.* mgm12 [m]; *M.* mgm13 [m].

### *Onychomyrmex* Emery

*O.* au02. [m: ANIC, CASENT0172370, examined based on images].

### *Paraprionopelta* Kusnezov

*Pa*. *minima* Kusnezov 1955 [m: CASENT0173342, not dissected].

### *Prionopelta* Mayr

*Pr. descarpentriesi* Santschi, 1924 [g: CASENT0081411]; *Pr.* mg01 [g: CASENT0218100]; *Pr.* mgm01 [m: CASENT0114588]; *Pr.* mgm02 [m: CASENT0218101]; *Pr.* mgm03 [m: CASENT0007320]; *Pr.* mgm04 [m: CASENT0113575]; *Pr.* mgm05 [m]; *Pr.* mgm06 [m]; *Pr.* mgm07 [m].

### *Stigmatomma* Roger

#### Type material

*Stigmatomma denticulatum* Roger, 1859. Holotype. Worker. Griechenland (Greece): Monte Scapo (Mt. Scapo), Insel Zante (Zante Is.). Von Kiesenwelter. [Museum für Naturkunde der Humboldt-Universität Berlin (ZMHB): 18992, CASENT0104566. Examined based on images. Only one specimen from Greece is in ZMHB and it is labeled “typus.” Original labels on this specimen seem to have been replaced by new labels. The new label mistakenly lists the collector as “Roger, S.” instead of Von Kiesenwelter.]

#### Malagasy material

*S.* mg01 [g: CASENT0083104]; *S.* mgm01 [m: CASENT0111612]; *S.* mgm02 [m: CASENT0109221]; *S.* mgm03 [m: CASENT0007087]; *S.* mgm04 [m: CASENT0059491].

#### Non Malagasy material

*S. pallipes* (Haldeman, 1844) [w]; *S. denticulatum* (Roger, 1859) [w].

### *Xymmer* Santchi

#### Type material

*Stigmatomma* (*Xymmer*) *muticum* Santchi 1914. Lectotype [here designated]. Worker. NIGERIA: Nigerie S. lbadan (Silvestri). Det. Santchi 1913 [The Naturhistorisches Museum Basel (NHMB): CASENT0217322 not dissected)].

#### Non type Malagasy material

*X*. mgm01 [m: CASENT0083010]; *X*. mgm02 [m: CASENT0052348]; *X*. mgm03 [m: CASENT0218103]; *X*. mgm04 [m: CASENT0113147]; *X*. mgm05 [m: CASENT0080666]; *X*. mgm06 [m: CASENT0083494]; *X*. mgm07 [m]; *X*. mgm08 [m]; *X*. mgm09 [m].

#### Non type material from other than the Malagasy region (not dissected)

In addition to above, several undetermined male specimens were available from Central African Republic (*X*. cf01: CASENT0087255), Gabon (*X*. gam01: CASENT0247582), and Thailand (*X*. th01: CASENT0119328); *X. mutica* worker specimens were available from Ivory Coast (CASENT0006952), Cameroon (CASENT0009095), Central African Republic (CASENT0417121), and Gabon (CASENT0004308).

### Terminology

Overall, our preference is for terms used generally in Hymenoptera over terms uniquely applied within Formicidae for homologous characters. Morphological terminology follows our previous work ([3]: figures 1, 2; [4]: figures 1–21, 25–34; [5]: figures 1–3, 6, 11, 16–23, 34, 39, 40, 46–61, 76–81) and is based on Snodgrass [20], Gauld and Bolton [21], Bolton [22], and Huber and Sharkey [23]. Use of the term pygostyle follows Snodgrass [24] and basimere and harpago follows Snodgrass [25]; terminology of wing venation follows Wootton [26], Gauld and Bolton [21], and Serna *et al.* [27]; and specialized conical setae for anterior clypeal projections follows Ward [28]. The applications of these terms in the Amblyoponinae are illustrated in Figures 1, 2, 3, 4, 5, 6, 7, 8, 9, 10, 11, 12, 13 and 14.

**Figure 2:**
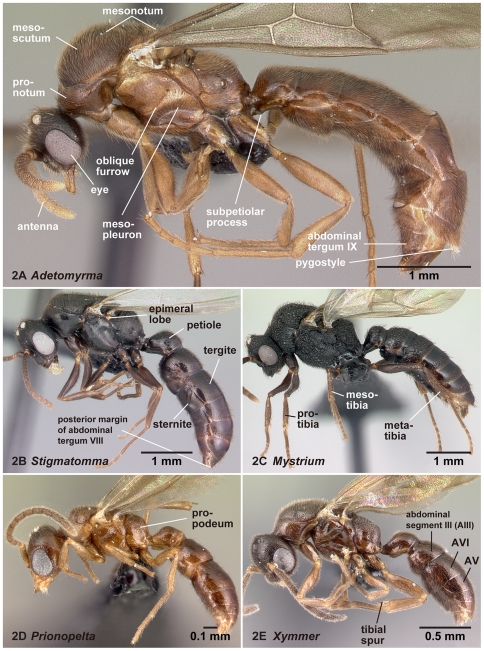
Males of the subfamily Amblyoponinae in lateral view. 2A, *Adetomyrma* mg02 (CASENT0079552); 2B, *Stigmatomma* mg01 (CASENT0080397); 2C, *Mystrium* mgm07 (CASENT0081390); 2D, *Prionopelta* mgm05 (CASENT0049679); 2E, *Xymmer* mgm06 (CASENT0083484).

**Figure 3:**
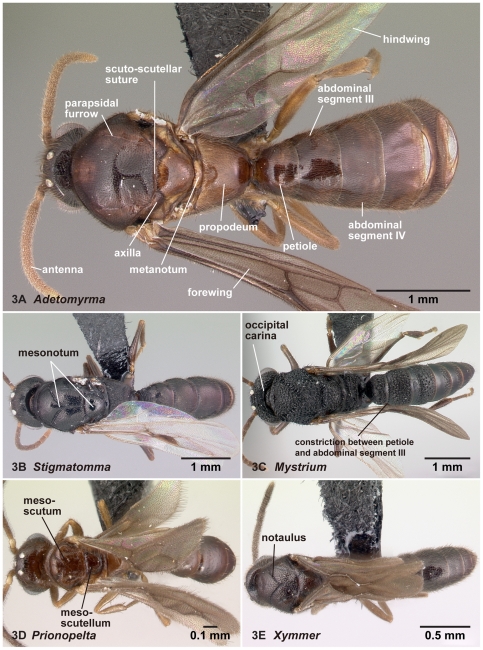
Males of the subfamily Amblyoponinae in dorsal view. 3A, *Adetomyrma* mg02 (CASENT0079552); 3B, *Stigmatomma* mg01 (CASENT0080397); 3C, *Mystrium* mgm07 (CASENT0081390); 3D, *Prionopelta* mgm05 (CASENT0049679); 3E, *Xymmer* mgm06 (CASENT0083484).

**Figure 4:**
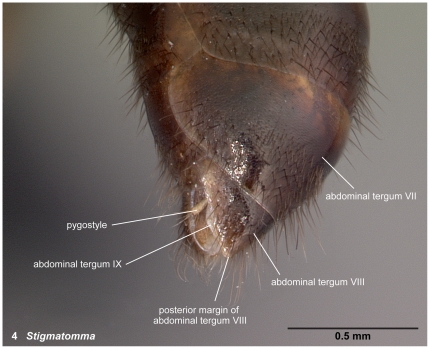
Posterior portion of the abdomen in oblique dorsal view. 4, *Stigmatomma* mg01 (CASENT0080397).

**Figure 5:**
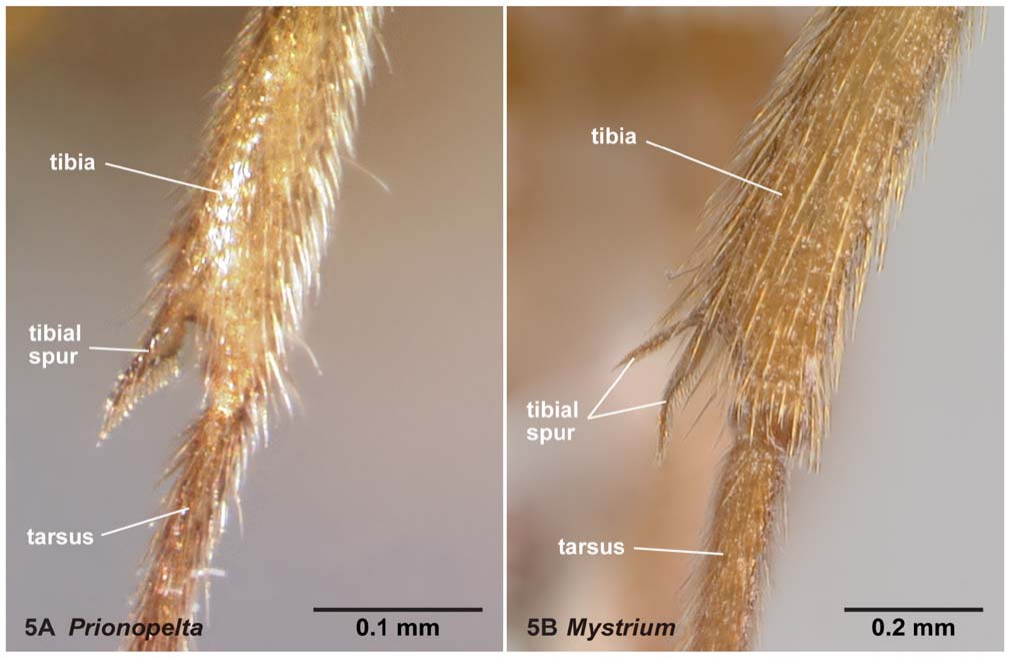
Left metatibia in frontal view. 5A, *Prionopelta* mg01 (CASENT0049809); 5B, *Mystrium* mg05 (CASENT0492154).

**Figure 6:**
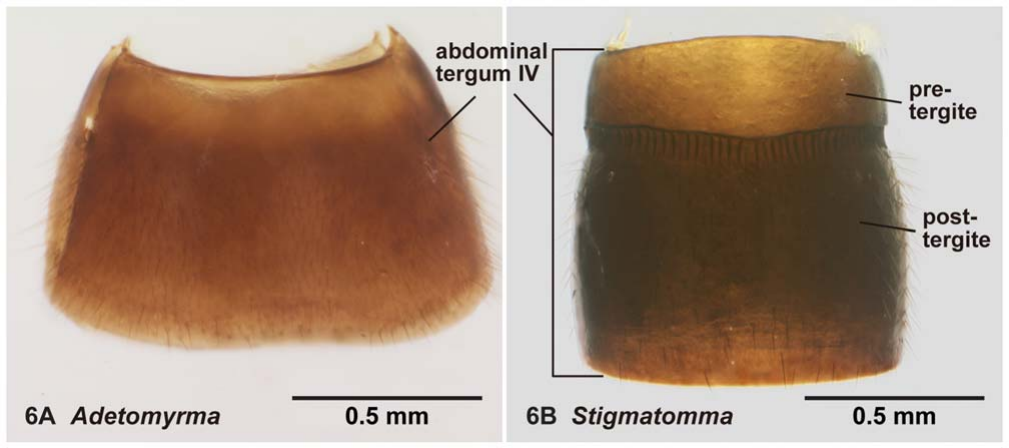
Abdominal tergum IV in dorsal view. 6A, *Adetomyrma* mg02 (CASENT0218011); 6B, *Stigmatomma* mg01 (CASENT0083104).

**Figure 7:**
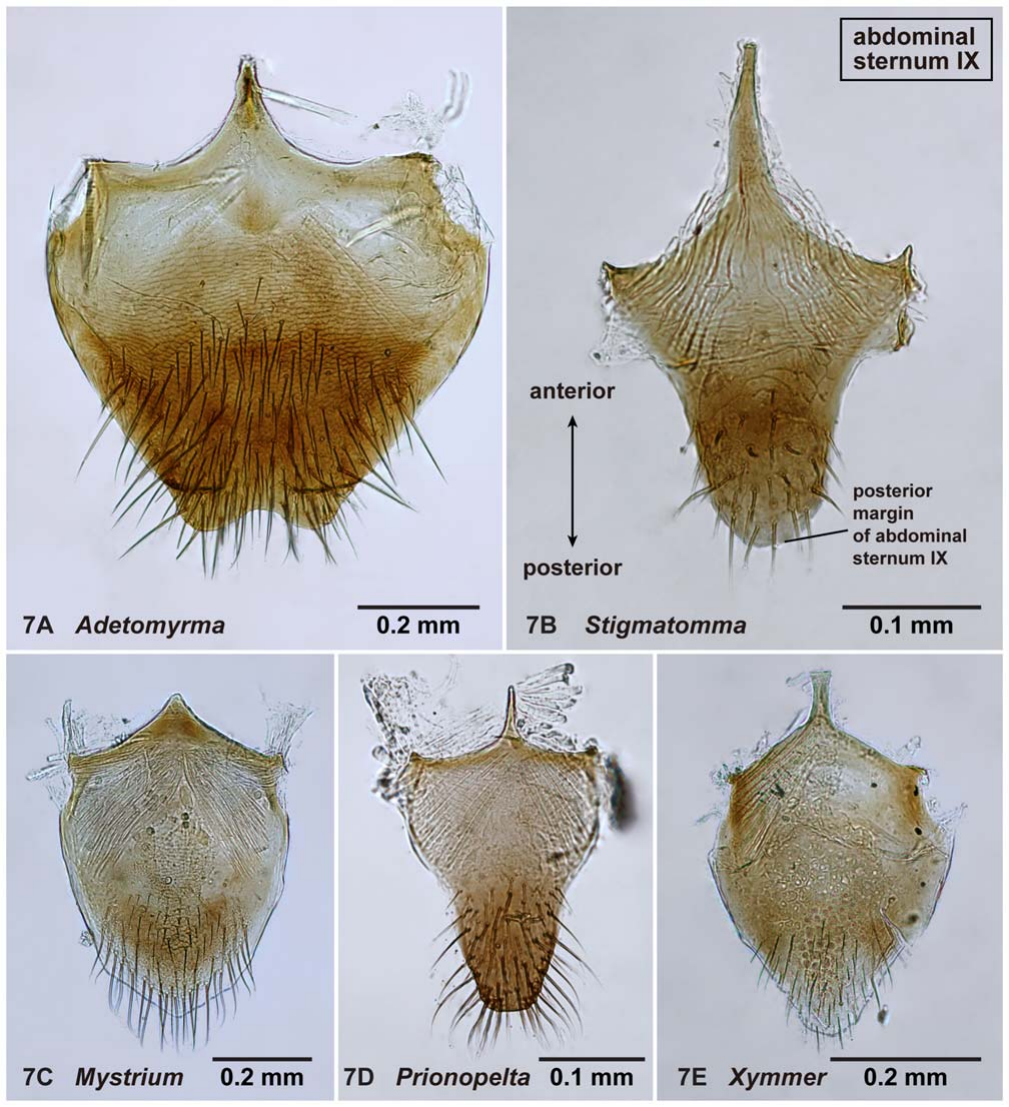
Abdominal sternum IX of amblyoponine males in ectal view. 7A, *Adetomyrma* mg02 (CASENT0218011); 7B, *Stigmatomma* mgm01 (CASENT0111612); 7C, *Mystrium* mgm02 (CASENT0078803); 7D, *Prionopelta descarpentriesi* (CASENT0081411); 7E, *Xymmer* mgm02 (CASENT0052348).

**Figure 8:**
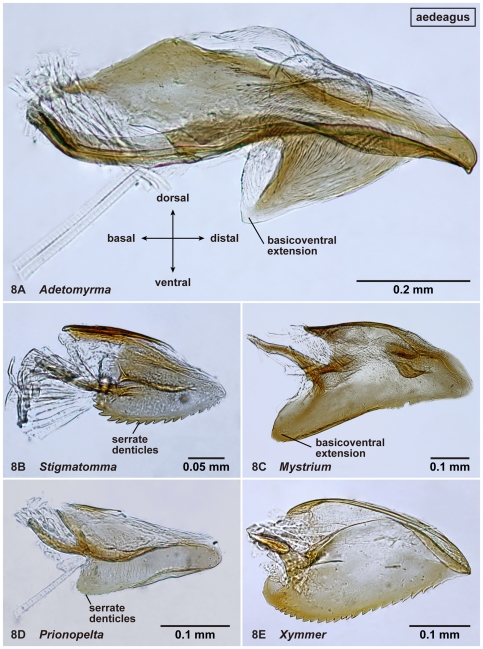
Right aedeagus of amblyoponine males in mesal view. 8A, *Adetomyrma* mg02 (CASENT0218011); 8B, *Stigmatomma* mgm01 (CASENT0111612); 8C, *Mystrium* mgm02 (CASENT0078803); 8D, *Prionopelta descarpentriesi* (CASENT0081411); 8E, *Xymmer* mgm02 (CASENT0052348).

**Figure 9:**
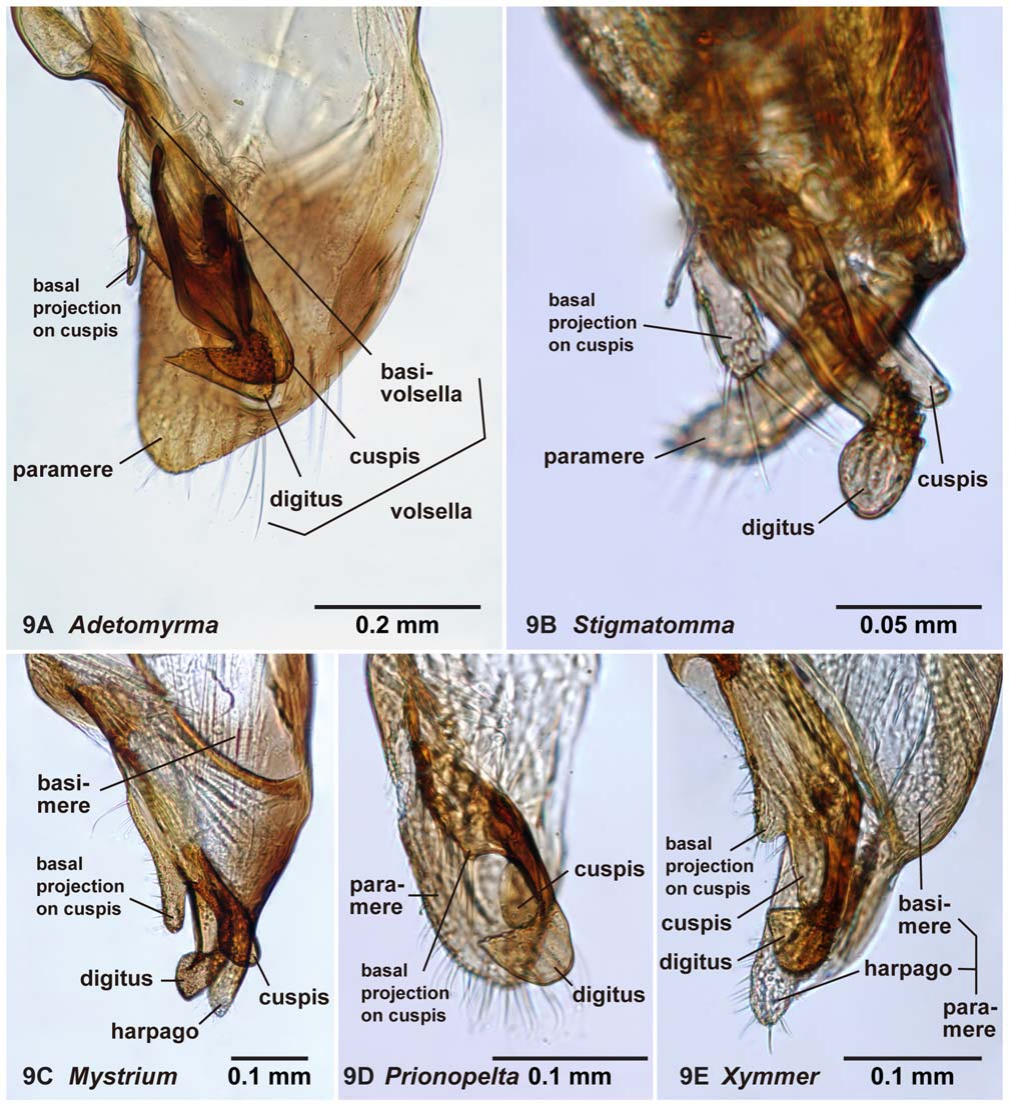
Right volsella of amblyoponine males in mesal view. 9A, *Adetomyrma* mg02 (CASENT0218011); 9B, *Stigmatomma* mgm01 (CASENT0111612); 9C, *Mystrium* mgm02 (CASENT0078803);9 D, *Prionopelta descarpentriesi* (CASENT0081411); 9E, *Xymmer* mgm02 (CASENT0052348).

**Figure 10:**
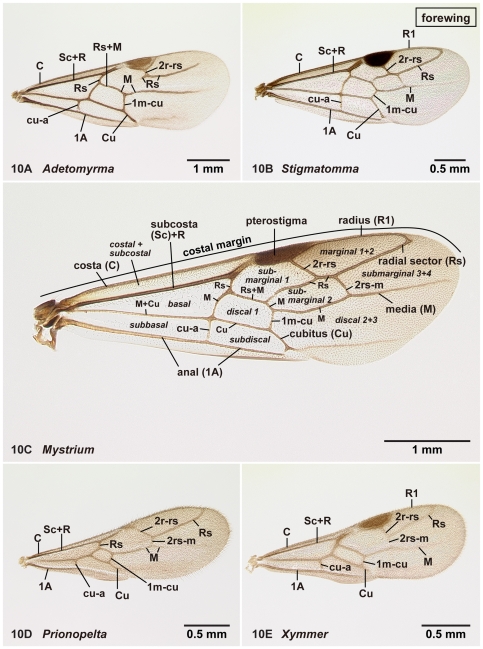
Right forewing of amblyoponine males in dorsal view. 10A, *Adetomyrma* mg05 (CASENT0218013); 10B, *Stigmatomma* mg01 (CASENT0083104); 10C, *Mystrium rogeri* (CASENT0218102); 10D, *Prionopelta descarpentriesi* (CASENT0081411); 10E, *Xymmer* mgm04 (CASENT0113147).

**Figure 11:**
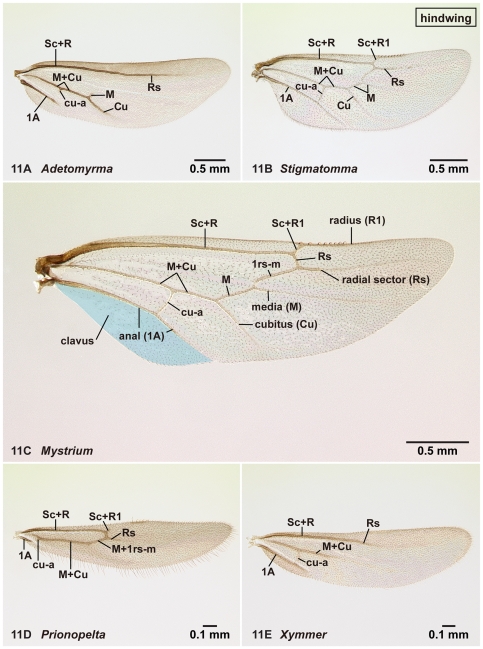
Right hindwing of amblyoponine males in dorsal view. 11A, *Adetomyrma* mg05 (CASENT0218013); 11B, *Stigmatomma* mg01 (CASENT0083104); 11C, *Mystrium rogeri* (CASENT0218102); 11D, *Prionopelta descarpentriesi* (CASENT0081411); 11E, *Xymmer* mgm04 (CASENT0113147).

**Figure 12:**
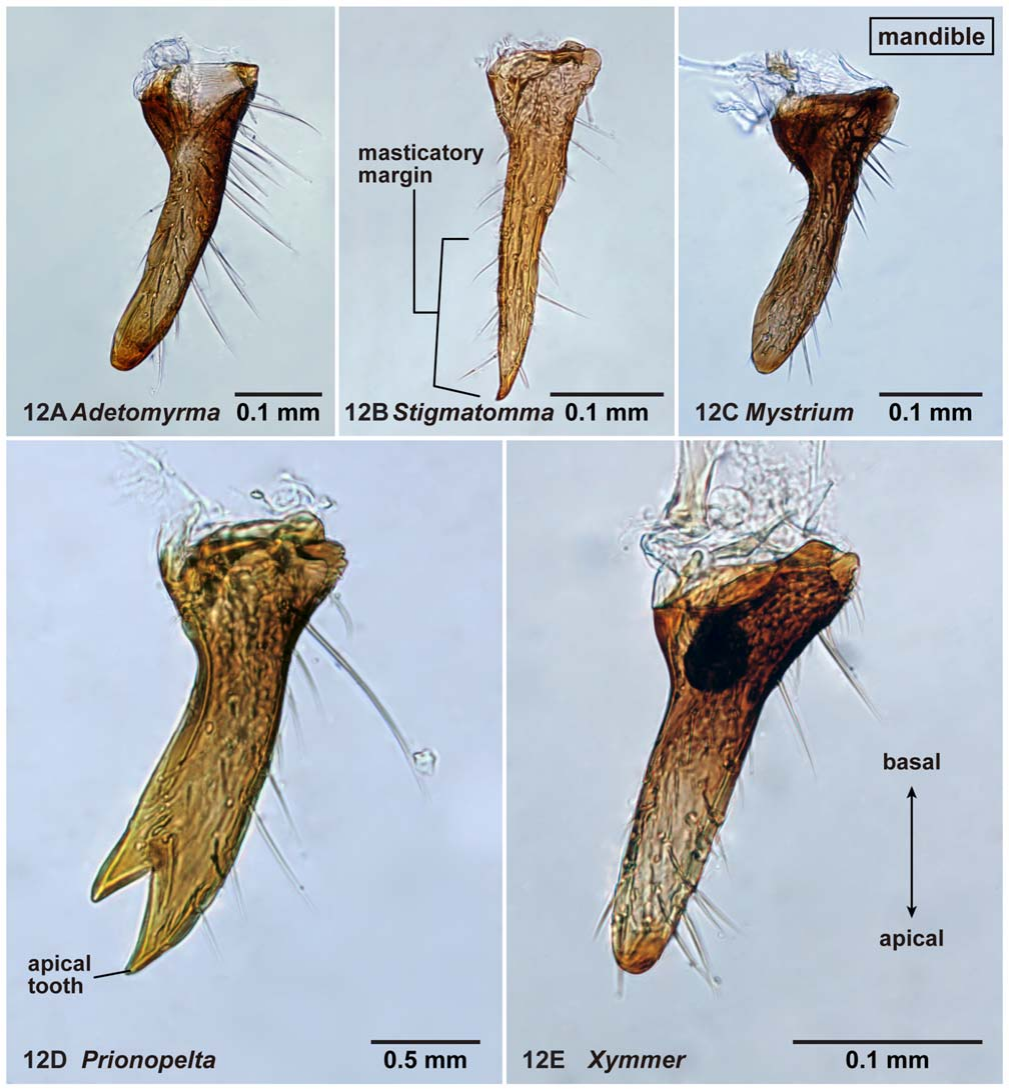
Left mandible of amblyoponine males in oblique dorsal view. 12A, *Adetomyrma* mg02 (CASENT0218011); 12B, *Stigmatomma* mgm03 (CASENT0007087); 12C, *Mystrium* mgm02 (CASENT0078803); 12D, *Prionopelta descarpentriesi* (CASENT0081411); 12E, *Xymmer* mgm02 (CASENT0052348).

**Figure 13:**
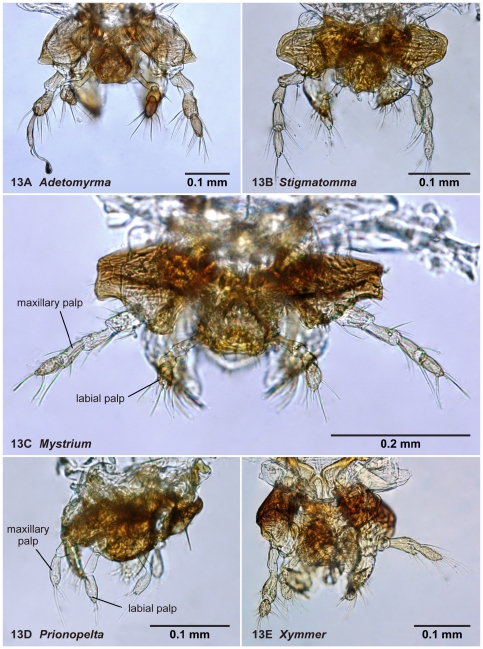
Mouthparts of amblyoponine males in anterior view. 13A, *Adetomyrma* mg02 (CASENT0218011), an anomaly is observed on the left maxillary palp; 13B, *Stigmatomma* mgm03 (CASENT0007087); 13C, *Mystrium* mgm02 (CASENT0078803); 13D, *Prionopelta descarpentriesi* (CASENT0081411); 13E, *Xymmer* mgm02 (CASENT0052348). Each image is modified to clearly show its palpal segments.

**Figure 14:**
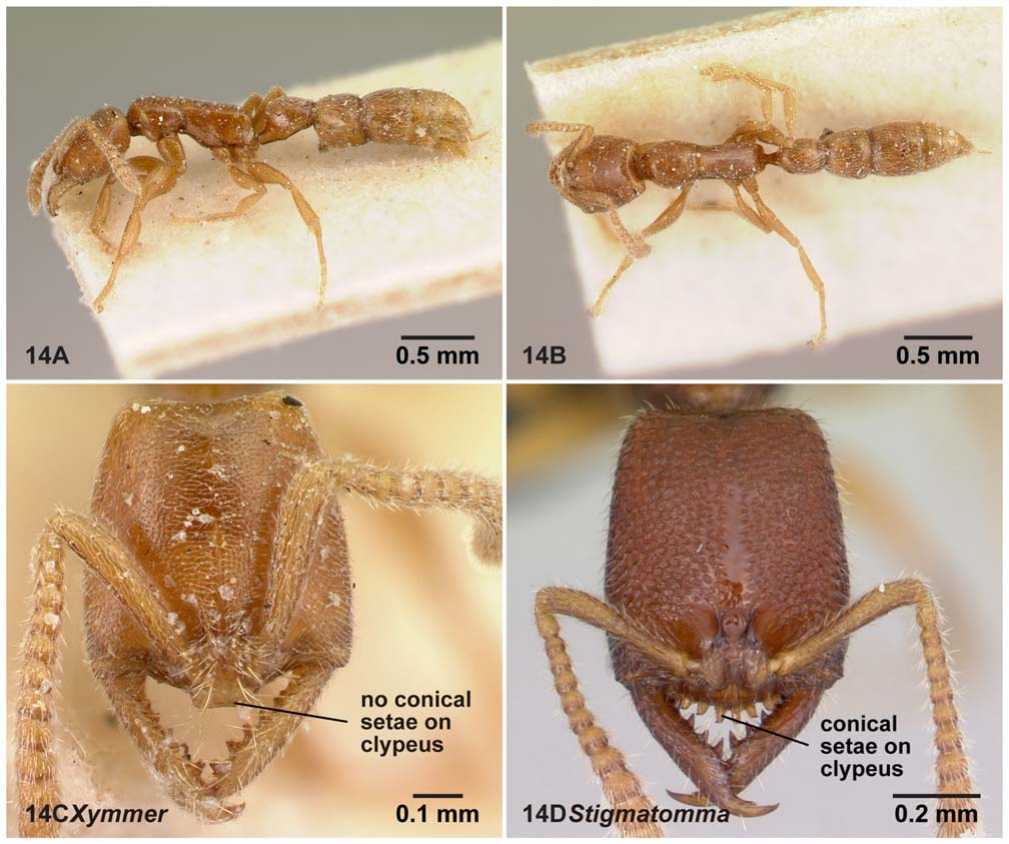
Workers of *Xymmer* and *Amblyopone*. 14A–C, *Xymmer muticus* (CASENT0217322: lectotype, designated in this study); 14D, *Stigmatomma* mg06 (CASENT0022237). 14A, lateral view; 14B, dorsal view; 14C, 14D, full-face view.

#### Tergosternal fusion

In this study, tergosternal fusion is considered to occur when tergite and sternite are fused to each other in a manner different from the fusion seen in the proceeding posterior segment. The degree of fusion in males seems to be highly variable, with many intermediates. According to Bolton [29], the tergosternal fusion seen in abdominal segments III and IV was pointed out in Gotwald [30] for the first time. Bolton classified segments as “fused” when “the tergite and sternite of any given segment either meet edge to edge, or narrowly overlap and are immovably welded together.” In the Amblyoponinae the level of fusion is difficult to ascertain, especially when the segments overlap. Therefore, we use a relative comparison with abdominal segment V which is never fused in ants.

#### Abdominal sternum IX (Figure 7)

We prefer the term abdominal sternum IX rather than the subgenital plate and the hypopygium, the terms used for this sclerite in previous studies, because the latter two terms are ambiguous in homology; the subgenital plate and the hypopygium are not homologous in males and females of the same taxon. Even within the same sex, the subgenital plate is not consistent between taxa. (More detailed information is provided in the terminology section and table 1 of Yoshimura & Fisher [5]).

**Table 1:** Character matrix for males of Malagasy Amblyoponinae.

Characters	01	02	03	04	05	06	07	08	09	10	11	12	13	14	15
*Adetomyrma*	1(9)	1(9)	1(9)	2(2)/3(7)	2(1)/3(8)	13(9)	1(8)/0(1)	1(5)/0(3)	0(9)	2(9)	2(9)	1(9)	1(9)	0(9)	1(3)/0(5)
*Mystrium*	0(12)	1(11)	1(11)	4(10)	3(9)	13(12)	1(8)/0(3)	0(12)	0(8)	2(2)/1(9)	2(12)	0(12)	0(12)	0(8)	0(8)
*Prionopelta*	0(10)	1(9)	0(8)	2(7)	2(7)	13(9)	0(9)	1(8)	0(6)	1(8)	1(9)	0(9)	0(10)	0(6)	0(6)
*Stigmatomma*	1(5)	1(5)	1(5)	4(4)/3(1)	3(2)/2(3)	13(5)	0(5)	0(4)	0(5)	2(2)/1(3)	2(5)	0(5)	0(5)	0(5)	0(5)
*Xymmer*	1(9)	0(9)	1(9)	4(2)/3(7)	3(4)/2(5)	13(9)	0(9)	0(9)	0(6)	1(1)/0(8)	2(9)	0(9)	0(9)	0(6)	0(6)

Characters	16	17	18	19	20	21	22	23	24	25	26	27	28	29	30
*Adetomyrma*	0(9)	1(9)	1(2)/0(7)	1(9)	1(9)	2(7)/0(1)	0(9)	1(9)	1(9)	0(9)	1(9)	0(9)	1(9)	1(3)/0(6)	1(6)/1(3)
*Mystrium*	1(12)	0(8)	1(8)	1(8)	0(8)	1(8)	0(8)	0(8)	0(8)	0(8)	0(8)	1(4)/0(4)	0(8)	0(8)	0(8)
*Prionopelta*	0(9)	0(6)	1(6)	1(3)/0(3)	0(6)	0(6)	1(6)	1(6)	0(6)	1(6)	0(6)	0(6)	0(6)	0(6)	1(6)
*Stigmatomma*	0(5)	1(2)/0(3)	1(4)/0(1)	1(5)	0(5)	0(5)	0(5)	0(5)	0(5)	0(5)	0(5)	1(2)/0(3)	0(5)	0(5)	1(2)/0(3)
*Xymmer*	1(9)	0(6)	1(6)	1(6)	0(6)	0(6)	0(6)	1(6)	0(6)	0(7)	0(6)	1(1)/0(5)	1(6)	1(7)	1(7)

For the 30 characters which seem useful for distinguishing among amblyoponine genera, character states are shown 0, 1, 2, exact number, or as 0/1 (if both states 0 and 1 were observed for each genus). The number of species in which the character states were observed is given in parentheses following the character state. Character states have been confirmed by direct observation or by dissection.

#### Pygostyles (Figure 4)

Pygostyle is used to refer to the pair of appendages on tergum X of the abdomen of male Hymenoptera. Cerci, on the other hand, should refer to appendages on abdominal tergum XI, not X (see also table 1 in Yoshimura & Fisher [5] and Yoshimura & Fisher [3]).

#### Venation and cells on the wings (Figures 10, 11)

We use veins, not cells, to describe wing characters and to discuss the homology of wing characters between amblyoponine genera and genera in other subfamilies. In previous studies of Amblyoponinae [6], [8], [12] other than Brown's works [9], [17], the number of closed cells on the fore- and hindwings was used to highlight differences between taxa. The focus on cells, however, diverts attention away from careful comparative studies of venation patterns as mentioned in Brown & Nutting [31] (see also table 1 in Yoshimura & Fisher [4] and the terminology section in Yoshimura & Fisher [5]).

In the present study, the homology of each vein across taxa was determined based on comparative studies of taxa with well-developed veins, such as species in the genus *Mystrium* (Figures 10C, 11C). Terms used to define veins follow the recommendation of Wootton [26], Gauld and Bolton [21], and Serna *et al*. [27]. However, we use 1A on the forewing instead of A; 1rs-m on the hind wing instead rs-m. Our naming system for wing veins is summarized in Figures 10C (forewing) and 11C (hindwing). Cell names as indicated in Gauld and Bolton [21] are also given in Figure 10C. We provide cell names only in reference to this earlier study, and not as an endorsement for their use.

The names of wing cells in ants have been inaccurately interpreted due to homonymy. Submarginal cells 1 and 2 in Figure 10C were referred to as cubital cells in previous works for Amblyoponinae [6], [8], [12], [17]; however, cells formed between cubitus and anal, i.e. subbasal and subdiscal in Figure 10C, were also referred to as 1st and 2nd cubital cells by Huber and Sharkey [23]. Nichols defines the cubital cells as wing cells bounded anteriorly by the cubitus or one of its branches [32], and agrees with the naming system by Huber and Sharkey [23]. On the other hand, “cubital cells” in Amblyoponinae [6], [8], [12], [17] are bounded anteriorly by subcosta and pterostigma, or Rs+M and radial sector, demonstrating the inconsistent application of the term “cubital cell(s)” in ant taxonomy.

Serna *et al*. [27] revised wing cell names proposed in their previous paper [33]. Their new system, however, still disagrees with Gauld and Bolton's system in some areas. For example, Serna *et al.* [33] used the terms submarginal cell 3 and discal cell 2 to identify forewing cells instead of marginal 1+2 and subdiscal 1 respectively, as in Gauld and Bolton [21]. According to Nichols's definition [32], cells immediately distal to pterostigma and bordering the costal margin are marginal cells. The marginal cells in Gauld and Bolton [21] are identical with the Nichols's definition, but the one in Serna *et al*. [27] is not. Creating new homonymies in terminology is not preferred because it could lead to more confusion in future comparative studies. Therefore, marginal 1+2 and subdiscal 1 should be used instead of submarginal cell 3 and discal cell 2 in the sense of Serna *et al.* [33].

### Morphological analysis

**Figure 15:**
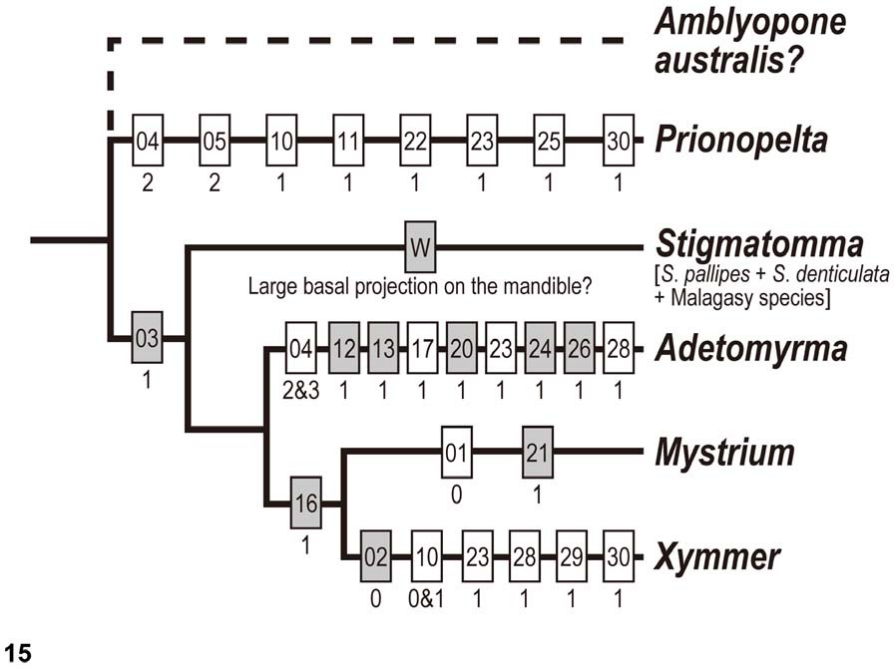
Morphological characters mapped on phylogenetic topology of XMAS clade and *Prionopelta* as an outgroup. Characters plotted on the topology of the XMAS presented in Brady *et al*. [13]. Numbers in each box correspond to character in Table 1; numbers under the box represent character states; boxes filled in gray indicate those characters are unique to each genus or clade; dashed line is hypothetical.

A detailed morphological examination was carried out for 30 male characters. Some characters were chosen based on their relevance in previous studies: e.g., the presence of the pygostyles as cerci [6], the number of the mesotibial spurs [7], the presence of radial sector and position of cu-a [17], and the fusion of the metacoxal cavity [28]. Morphological characters examined are listed below. Results of the examination are given as a character matrix (Table 1) and used for exploring character evolution (Figure 15: see discussion). A key and diagnoses for genera in the Malagasy region based on the matrix are provided.

### A list of characters

01. Frontal carinae are absent (1); present (0)

02. Dent-like projections are present on anterior margin of the clypeus (1); no such projections on the margin (0)

03. The number of mandibular teeth (Figure 12)

04. The number of maxillary palpal segments (Figure 13)

05. The number of labial palpal segments (Figure 13)

06. The number of antennal segments

07. Notaulus absent on the mesoscutum (1: as in Figure 3A); present on the mesoscutum (0: as in Figure 3B)

08. Epimeral lobe absent (1: as in Figure 2A); present (0: as in Figure 2B)

09. Metacoxal cavity completely fused (1); touches but is not fused (0)

10. The number of mesotibial spurs

11. The number of metatibial spurs (Figure 5)

12. In dorsal view, constriction between AII (petiole: the second abdominal segment) and AIII absent (1: as in Figure 3A); present (0: as in Figure 3C)

13. A transverse furrow dividing pre- and post-sclerite on AIV absent (1: as in Figure 6A); present (0: as in Figure 6B)

14. Tergosternal fusion not found on AIII (1); found on AIII (0)

15. Tergosternal fusion not found on AIV (1); found on AIV (0)

16. Pygostyles absent (1); present (0: as in Figure 4)

17. Distal margin of abdominal sternum IX concave (1: as in Figure 7A); convex (0: as in Figure 7B)

18. Harpago separated from the basimere by a suture (1); separation between the harpago and basimere unclear (0)

19. Basal projection on the cuspis present (1: as in Figure 9); absent (0)

20. Ventral serrate teeth on the aedeagus absent (1: as in Figure 8A); present (0: as in Figure 8B)

21. Basicoventral portion of aedeagus sharply extended ventrally (2: as in Figure 8A); roundly extended basally (1: as in Figure 8C); not extended (0: as in Figure 8B)

22. On the forewing, the pterostigma is reduced in size (1: as in Figure 10D); is well developed (0: as in Figure 10A)

23. On the forewing, radial sector is wholly or partially absent between M+Rs and 2r-rs (1: as in Figures 10A, D, E); is complete on this portion (0: as in Figure 10B, C)

24. On the forewing, radial sector is not connected with radius (1: as in Figure 10A); is connected with radius (0: as in Figure 10B)

25. On the forewing, 2r-rs is connected with radial sector far distal from the pterostigma (1: as in Figure 10D); is connected with radial sector posterior to the pterostigma (0: as in Figures 10A–C, D)

26. On the forewing, 2rs-m is absent (1: as in Figure 10A); present (0: as in Figure 10B)

27. On the forewing, cu-a is located at or close to a junction of media and cubitus (0: as in Figure 10B); is located far basal from the junction (0: as in Figure 10A)

28. On the hindwing, 1rs-m is absent (1: as in Figures 11A, E); is present (0: as in Figures 11B–D)

29. On the hindwing, media is absent between 1rs-m and cubitus (1: as in Figure 11E); is present on this portion (0: as in Figures 11A–D)

30. On the hindwing, media is absent apical to 1rs-m (1: as in Figures 11A, D, E); is present on this portion (0: as in Figures 11B, C)

### Nomenclatural acts

The electronic version of this document does not represent a published work according to the International Code of Zoological Nomenclature (ICZN), hence the nomenclatural acts contained in the electronic version are not available under that Code from the electronic edition [34]. Therefore, a separate edition of this document was produced by a method that assures numerous identical and durable copies, and those copies were simultaneously obtainable (from the publication date noted on the first page of this article) for the purpose of providing a public and permanent scientific record, in accordance with Article 8.1 of the Code [34]. The separate print-only edition is available on request from PLoS by sending a request to PLoS ONE, Public Library of Science, 1160 Battery Street, Suite 100, San Francisco, CA 94111, USA, along with a check for $10 (to cover printing and postage) payable to “Public Library of Science.”

## Results

Results of the detailed morphological examination of males of five Malagasy genera *Adetomyrma*, *Mystrium*, *Prionopelta*, *Stigmatomma*, and *Xymmer*, based on 30 characters. The criteria used are given as a character matrix (Table 1).

### Diagnosis of males of the subfamily Amblyoponinae in the Malagasy region

Males alate. Scape not reaching posterior margin of head (Figure 1). Mesopleural oblique furrow usually vestigial, and when present, reaching anterior margin of mesopleuron far ventrally from posteroventral corner of pronotum (Figure 2). Notaulus present (Figures 3B–E) or absent (Figure 3A). Scuto-scutellar suture simple or with longitudinal sculpture. Metacoxal cavity encircled by cuticle, endpoints meeting broadly but not fused. Protibia with single, well-developed spur. Mesotibia with one to two spurs or without spur. Metatibia with one to two spurs (Figure 5). Petiole attached to abdominal segment III dorsally, so that dorsal margin of petiole higher than or same height as dorsal margin of abdominal segments III in lateral view. Abdominal segment III larger than or same size as segment IV (Figure 2). Constriction present (Figure 3A) or absent (as in Figures 3B, C) between abdominal segments III and IV. With abdominal segment IV in lateral view, its dorsal margin as long as its ventral margin. Abdominal segment III with tergosternal fusion. Abdominal segment IV with tergosternal fusion except in some species of *Adetomyrma*. The distal margin of abdominal sternum IX either convex or concave, but never bispinose. Pygostyles present (Figure 4) or absent. Basimere not distinctly differentiated from harpago, separation between basimere and harpago sometimes unclear (Figure 9). Volsella directed ventrally, but never stout and claw-shaped nor extended dorsally. A process present on the basal portion of cuspis in most species (Figure 9).

Venation on forewing and hindwing varies. On forewing (Figure 10), costa (C), Sc+R, Media (M), cubitus (Cu), anal (1A), 1m-cu and cu-a present in all genera. On hindwing (Figure 11), Sc+R, M+Cu and anal (1A) present. Clavus developed to reduced in size, and jugum absent.

#### Remarks

We propose that the process on the basal portion of the cuspis on the volsella (Figure 9) is a character unique to Amblyoponinae. It cannot be used as a complete diagnostic character due to the reduction of this process in some males of *Prionopelta*. The presence of this process has been illustrated and/or mentioned in several previous studies for *Amblyopone* sensu Brown (1960) (e.g. figure 24 A in Brown [9]; figure 21 in Gotwald [35]; figure 9 in Ogata [11]), and for *Myopopone* (diagnosis and figure 13 in Brown [9]). We confirmed the absence of this process in Proceratiinae and Ponerinae; these data and a polymorphism in *Prionopelta* suggest that reductions of this process in some, but not all, *Prionopelta* constitute a case of secondary loss.

The above character, which is difficult to confirm without dissection, is the only one unique to the subfamily Amblyoponinae. However, combinations of characters under diagnosis clearly separate the Amblyoponinae from the six other subfamilies in the Malagasy region. In the Malagasy region, Amblyoponinae differ from Cerapachyinae in lacking a bispinose abdominal sternum IX (Figure 7) and in having a moderate volsella; from Dolichoderinae in having a broader and higher attachment of the petiole to abdominal segment III and poorly differentiated basimere compared with the harpago; from Formicinae in having the petiole as above and the shorter scape of the antenna never reaching the posterior margin of the head (Figure 1); from Myrmicinae and Pseudomyrmecinae in having a slightly reduced or same-sized abdominal segment III compared with IV (as in Figure 2); from Ponerinae in having a broader and higher attachment of the petiole to abdominal segment III; and from Proceratiinae in having the dorsal margin of abdominal segment IV nearly the same length as its ventral margin in lateral view (in Proceratiinae, the dorsal margin of abdominal segment IV in lateral view is at least twice as long as its ventral margin) (Figure 2).

Amblyoponinae males have a broad, dorsal attachment of the petiole to abdominal segment III. However, this character is sometimes difficult to differentiate from the attachment in Proceratiinae, which may also appear broadly attached. The differences in the shape of abdominal segment IV mentioned above, however, will easily separate the two subfamilies.

### Key to genera of males of Amblyoponinae in the Malagasy region

This key may not apply outside of the Malagasy region, as variations in genus-level characters elsewhere have not been fully explored.

1. A single tibial spur present on hind leg (Figure 5A). Mandible with two distinct teeth (Figure 12D). Pterostigma reduced in size (Figure 10D)… *Prionopelta*

-. Two tibial spurs present on hind leg (Figure 5B). Mandible with a single apical tooth (Figures 12A–C, E). Pterostigma well developed (Figures 10A–C, E)… 2

2. Constriction between petiole and abdominal segment III indistinct in dorsal view (Figure 3A). Pretergite of abdominal segment IV not divided from posttergite by transverse furrow (Figure 6A). On forewing, radial sector fails to reach costal margin and is disconnected from radius (Figure 10A)… *Adetomyrma*

-. Constriction between petiole and abdominal segment III distinct in dorsal view (Figure 3C). Pretergite of abdominal segment IV distinctly divided from posttergite by transverse furrow (Figure 6B). On forewing, radial sector reaches costal margin and is connected with radius (Figures 10B, C, E)… 3

3. Pygostyles present (Figure 4)… *Stigmatomma*

-. Pygostyles absent… 4

4. Anterior margin of clypeus with dent-like projections (Figure 1C). On forewing, radial sector complete (Figure 10C). On hindwing, radius present (Figure 11C)… *Mystrium*

-. Anterior margin of clypeus without dent-like projections (Figure 1E). On forewing, radial sector wholly or partially absent between M+Rs and 2r-rs (Figure 10E). On hindwing, radius absent (Figure 11E)… *Xymmer*

### Diagnoses of males of extant genera of Amblyoponinae in the Malagasy region

Descriptions and diagnoses apply to species found in Madagascar. Diagnostic characters uniquely observed in each genus are given in italics. Male diagnoses for the genus *Adetomyrma* and *Xymmer* are proposed for the first time.

***Adetomyrma*** **Ward, 1994.** (Figures 1A, 2A, 3A, 6A, 7A, 8A, 9A, 10A, 11A, 12A, 13A)

With characters of Amblyoponinae. Frontal carinae absent. Anterior margin of clypeus with dent-like projections. Antenna consisting of 13 segments. Mandible with single, blunt apical tooth (Figure 12A). Palpal formula 3,3/2,3/2,2 (one specimen each of nine morphospecies dissected: Figure 13A). Notaulus distinct or absent. Mesepimeron with or without distinct posterodorsal lobe (epimeral lobe). Mesotibia with two spurs in most cases, rarely with single spur. Metatibia with two spurs. *In dorsal view, no constriction present between petiole and abdominal segment III* (Figure 3A). Abdominal segment IV with or without tergosternal fusion. *Pretergite of abdominal segment IV not distinctly differentiated from posttergite, without transverse furrow between them* (Figure 6A). Pygostyles present.

Distal margin of abdominal sternum IX concave (Figure 7A). Separation between basimere and harpago usually indistinct but distinct in some species. Basal projection on cuspis well-developed (Figure 9A). Aedeagus in lateral view, its basicoventral portion extended basally in most cases, shape of extension somewhat triangular to subtriangular, with relatively sharper distal apex; *serrate denticles absent on basal portion of ventral margin of aedeagus* (Figure 8A).

On forewing (Figure 10A), pterostigma well-developed, radial sector wholly or partially absent between M+Rs and 2r-rs, *radial sector fails to reach costal margin*, 2r-rs connected with radial sector posterior to pterostigma, *2rs-m absent*, cu-a located far from junction between media and cubitus. On hindwing (Figure 11A), radius absent in most cases but rarely weakly developed, 1rs-m absent, media usually present apical to 1rs-m.

#### Additional genus characters other than generic diagnoses

In full-face view, head wider than long when eyes included, ocelli well-developed, eyes well-developed and protruding laterally, situated on middle to anterior portion of lateral margin of head (as in Figure 1A). Occipital carina absent. Anterior clypeal margin distinctly or weakly convex anteriorly (Figure 1A). Antenna stout and not extremely long, scape short, never reaching posterior margin of head in full-face view. Mandibles cross when fully closed.

Mesopleural oblique furrow very weak or absent, reaching anterior margin of mesopleuron far ventrally from posteroventral corner of pronotum when the furrow is visible (Figure 2A). With mesonotum in dorsal view, parapsidal furrows deeply impressed (Figure 3A); the axilla clearly divided. Propodeum without teeth or spines on its posterodorsal portion.

Subpetiolar process developed to various degrees, absent in several species. The distal margin of abdominal tergum VIII relatively flat, not strongly protruding on middle portion.

Body sculpture weak. Body color yellow to blackish brown.

#### Remarks

A male synopsis of the genus *Adetomyrma* is provided for the first time based on nine morphospecies. Genus *Adetomyrma* is endemic to Madagascar. Males of *Adetomyrma* are distinguished easily from the other four Malagasy amblyoponine genera by lack of a constriction between petiole and abdominal segment III in dorsal view (Figure 3A), lack of a transverse furrow dividing pre- and posttergite of abdominal segment IV (Figure 6A), radial sector on forewing failing to reach the costal margin, and absence of 2rs-m on forewing (Figure 10A).

Other than characters uniquely observed in *Adetomyrma*, absence of 1rs-m on the hindwing is shared with *Xymmer*.

In addition to the separable characters above, two additional characters can be useful to distinguish *Adetomyrma* from the other Malagasy amblyoponine males: the posterior margin of abdominal sternum IX shallowly to deeply concave (Figure 7A), and a triangular expansion on the aedeagus (Figure 8A). However, several exceptions to these features were observed. A convex posterior margin of abdominal sternum IX is common among amblyoponine males in this region, while a concave posterior margin is observed in only a few males of *Stigmatomma* other than *Adetomyrma*. A basicoventral, triangular-like expansion on the aedeagus is unique to *Adetomyrma*, and this character easily separates *Adetomyrma* from the other genera. However, only one large morphospecies, *Adetomyrma* mg03, has extraordinarily specialized genitalia, and the expansion in this species only is vestigial. The bizarre genital features in *Ad*. mg03 are not similar to any other known males in Amblyoponinae. A basicoventral expansion on the aedeagus is confirmed also in *Mystrium* (Figure 8C); however, the expansion in the latter has two differences from the former: 1) serrate dents are present on its ventral margin; and 2) distal margin of the expansion is rounded, never triangular.

New palpal formulae in the genus *Adetomyrma* were found in this study. We observed three palpal formulae in *Adetomyrma* males, 3,3 (Figure 13A)/2,3/2,2, although only one formula 3,3 has been reported in *Adetomyrma* based on workers (Ward [28], original description). According to Brown's observation, the palpal formula observed in males of *Amblyopone* sensu Brown [9] is frequently the same as that in conspecific workers [9]. We confirmed this tendency in *Mystrium* and *Prionopelta* as well through observation. Two other types of palpal formulae may be observed in the workers of *Adetomyrma* with further observation, although 3,3 is the most common.

**A lack of tergosternal fusion** in abdominal segment IV was observed only in *Adetomyrma* among amblyoponine males in the Malagasy region. Interestingly, we found polymorphisms in this character within *Adetomyrma*. Tergosternal fusions in abdominal segments II (petiole) to IV were focused on in Gotwald [30], and redefined by Bolton [29]. Since its discussion in Bolton [29], this character has been regarded as an important diagnostic character for poneromorph subfamilies (sensu Bolton [10]). Ward proposed the lack of tergosternal fusions in abdominal segments III and IV to be a probable case of generic autapomorphy in workers of *Adetomyrma* [28]. In this work, Ward raised an interesting question for ant systematics in the form of a pair of hypotheses: either a tergosternal fusion in ants occurred independently in several lineages, or represents a reversal. Saux *et al*. inferred that *Adetomyrma* had undergone secondary reversal of tergosternal fusion, based on the distribution of character states on their phylogenic tree [36]. In our observations, interestingly: 1) tergosternal fusion in abdominal segment III was confirmed in all males of morphospecies examined, although larger portions of tergite and sternite overlap more than in other amblyoponine males; 2) in one of these morphospecies (*Adetomyrma* mg02), the associated workers lacked tergosternal fusion in abdominal segment III; and 3) tergosternal fusion in abdominal segment IV is distinct in males of five of the nine morphospecies, while the other four are difficult to judge but also seem to lack tergosternal fusion in abdominal segment IV. The fact that the fusion of abdominal segment III is inconsistent between males and workers in at least one species suggests that the lack of tergosternal fusion in *Adetomyrma* workers is secondary. This result is consistent with the explanation proposed by Saux *et al.* [36].

***Mystrium*** **Roger, 1862.** (Figures 1C, 2C, 3C, 5B, 7C, 8C, 9C, 10C, 11C, 12C, 13C)

With characters of Amblyoponinae. Frontal carinae present. Anterior margin of clypeus with dent-like projections. Antenna consisting of 13 segments. Mandible with single, blunt, apical tooth (Figure 12C). Palpal formula 4,3 (one specimen each of eight morphospecies dissected: Figure 13C). Notaulus absent or indistinct in many cases, but distinct in several species. Mesepimeron with distinct posterodorsal lobe (epimeral lobe). Mesotibia with one or two spurs in most cases, rarely indistinct. Metatibia with two spurs (Figure 5B). In dorsal view, distinct constriction present between petiole and abdominal segment III (Figure 3C). Abdominal segment IV with tergosternal fusion. Pretergite of abdominal segment IV distinctly differentiated from posttergite; a deep transverse furrow divides them. Pygostyles absent.

Distal margin of abdominal sternum IX convex (Figure 7C). Separation between basimere and harpago distinct. Basal projection on cuspis well-developed (Figure 9C). *The basicoventral portion of aedeagus extended basally, distal margin of extension rounded* (Figure 8C). Aedeagus in lateral view, serrate denticles present on basal portion of its ventral margin.

On forewing (Figure 10C), pterostigma well-developed, radial sector fully present and reaches to costal margin, 2r-rs connected with radial sector posterior to pterostigma, 2rs-m present, cu-a position variable, located close to or far from junction between media and cubitus. On hindwing (Figure 11C), radius present, 1rs-m present, media present apical to 1rs-m.

#### Remarks

The only unique character of males of *Mystrium* found in this study was the basicoventral portion of the aedeagus roundly and strongly extending basally (Figure 8C) (see also the genus *Adetomyrma*). It is the first time a unique character for male *Mystrium* has been provided that distinguishes it from the other amblyoponine genera, although this character is difficult to observe without dissection.

In practice, males of *Mystrium* are distinguished easily from *Adetomyrma* by having a distinct constriction between the petiole and abdominal segment III (Figure 3C), from *Stigmatomma* by the lack of the pygostyle, from *Prionopelta* by having two metatibial spurs (Figure 5B), and from *Xymmer* by a complete radial sector on the forewing (Figure 10C).

In addition to a unique character in the aedeagus, the presence of clypeal dent-like projections, a palpal formula of 4,3, and a distinct epimeral lobe are provided as new diagnostic characters.

Several diagnostic characters provided in previous studies require updating. First, Emery described the notaulus in *Mystrium* as deeply impressed [6]; however, eight species/morphospecies out of the eleven examined lack the furrow. Second, Emery described the number of mesotibial spurs in *Mystrium* as two [6], and Wheeler used this number in his key to separate *Stigmatomma*, which had a single mesotibial spur [7]. However, our observations revealed the number of mesotibial spurs is not useful as a separable character at the genus rank in Amblyoponinae, because intra-generic variation was found in *Mystrium*, *Stigmatomma*, and *Xymmer* (see Character 10 in Table 1). In fact, Bolton recorded the number of mesotibial spurs in *Mystrium* as one [10]. Therefore, this character is not useful for distinguishing genera, although it may be useful at the species level.

***Prionopelta*** **Mayr, 1866.** (Figures 1D, 2D, 3D, 5A, 7D, 8D, 9D, 10D, 11D, 12D, 13D)

With characters of Amblyoponinae. Frontal carinae present. Anterior margin of clypeus with dent-like projections. Antenna consisting of 13 segments. *Mandible with two sharp teeth* (Figure 12D). Palpal formula 2,2, one specimen each of one species and five morphospecies dissected (Figure 13D). Notaulus distinct (Figure 3D). Mesepimeron without distinct posterodorsal lobe (epimeral lobe). Mesotibia with single spur. *Metatibia with single spur* (Figure 5A). In dorsal view, distinct constriction present between petiole and abdominal segment III. Abdominal segment IV with tergosternal fusion. Pretergite of abdominal segment IV distinctly differentiated from posttergite; a deep transverse furrow divides them. Pygostyles present.

Distal margin of abdominal sternum IX convex (Figure 7D). Separation between basimere and harpago distinct. Basal projection on cuspis usually well-developed, but reduced in size in several species (as in Figure 9D). Basicoventral portion of aedeagus not extraordinarily expanded (Figure 8D). Aedeagus in lateral view, serrate denticles present on basal portion of its ventral margin.

On forewing (Figure 10D), *pterostigma reduced in size*, radial sector absent between M+Rs and 2r-rs, radial sector reaches to costal margin, *2r-rs connected with radial sector distal to pterostigma*, 2rs-m present, cu-a located far from junction between media and cubitus. On hindwing (Figure 11D), radius present in most cases, but absent in one species, 1rs-m present, media present apical to 1rs-m.

#### Remarks

Males of *Prionopelta* are distinguished from the other Malagasy amblyoponine genera by having two teeth on the mandible (Figure 12D), a single metatibial spur (Figure 5A), the pterostigma reduced in size, and the 2r-rs connecting with the radial sector distal to the pterostigma (Figure 10D). The pterostigmal character is proposed as a new diagnostic character.

We found useful male-based generic characters of *Prionopelta* have been provided in three previous studies [8]–[10]. Brown included the male characters in a diagnosis of *Prionopelta* [9], Bolton listed several male characters in his Appendix 2 [10], and Kusnezov proposed several characters in his male key to separate *Prionopelta* from *Stigmatomma* (as *Ericapelta* Kusnezov) and *Paraprionopelta* Kusnezov [8]. From these studies, our observation confirmed 2,2 in palpal formula (Character 4 and 5 in Table 1) [8]–[10], two-toothed mandible (Character 3 in Table 1) [8], [9], presence of notaulus (Character 7 in Table 1) [9], single tibial spur (Character 11 in Table 1) [10], and wing venations on fore- and hindwings (Characters 22–30 in Table 1) [9] as useful generic characters.

Of these characters, the two-toothed mandible (Character 3) may not separate *Prionopelta* from several groups in another region (see discussion below).

Reduction in size of the basal projection on the cuspis is observed only in *Prionopelta* as an intra-generic variation, and seems to be the result of a reversal. Brown's diagnosis of *Prionopelta* included this reduction as a lack of a “second cusp.” We observed reductions in this projection in three males of Malagasy *Prionopelta*. However, we also observed the following: 1) all amblyoponine genera examined, other than *Prionopelta,* have a well-developed projection on the basal portion of the cuspis; 2) the distinct projection was observed even in males of Malagasy *Prionopelta* except in the three males mentioned above. These data suggest the occurrence of this reduction is limited to a portion of the genus *Prionopelta*.

***Stigmatomma*** **Roger, 1859 stat. rev.** (Figures 1B, 2B, 3B, 4, 6B, 7B, 8B, 9B, 10B, 11B, 12B, 13B, 14B, 16)

**Figure 16:**
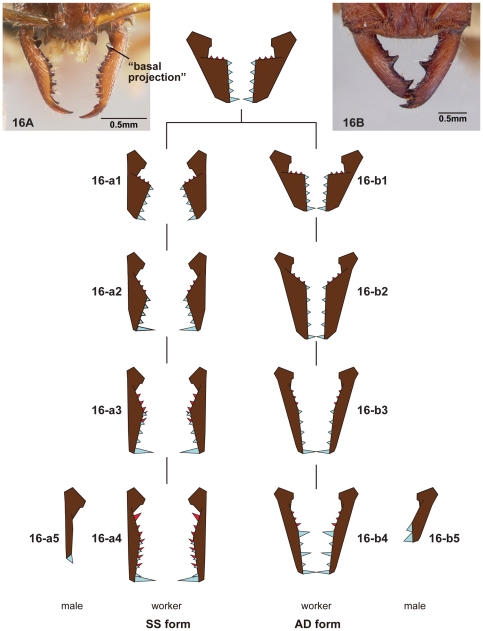
Mandibular transformation in *Stigmatomma* and *Amblyopone*. 16A, 16-a1-a5, Malagasy *Stigmatomma* (*Stigmatomma* mg01: CASENT0227519); 16B, 16-b1-b5, *Amblyopone australis* (CASENT0172268). 16A, 16-a1-a4, 16B, 16-b1-b4, worker mandible; 16-a5, 16-b5, male mandible. Series represents the transformations in workers' mandibles to SS form (16-a1-a5), with males having a single-toothed mandible, or AD form (16-b1-b5), with males having double-toothed mandible; mandibular teeth are blue and basal denticles are red.

*Stigmatomma* Roger, 1859: 250 [37], original description, type-species: *Stigmatomma denticulatum* Roger, 1859, by subsequent designation of Bingham, 1903: 36 [38] [Lectotype examined. See also Materials and Methods.]

*Arotropus* Provancher, 1881: 205 [39]. **syn. nov.**

*Fulakora* Mann, 1919: 279 [40].

*Lithomyrmex* Clark, 1928: 30 [41]. **syn. nov.**

*Ericapelta* Kusnezov, 1955: 273 [8]. **syn. nov.**

*Amblyopone* (in part): Brown, 1960 [9]; Bolton, 1995 [42]; 2003 [10].

With characters of Amblyoponinae. Frontal carinae absent. Anterior margin of clypeus with dent-like projections. Antenna consisting of 13 segments. Mandible with single, sharp, apical tooth (Figure 12B). Palpal formula 4,3/4,2/3,2 (one specimen each of five morphospecies dissected: Figure 13B). Notaulus distinct (Figure 3B). Mesepimeron with distinct posterodorsal lobe (epimeral lobe) in most cases, but rarely indistinct. Mesotibia with one or two spurs. Metatibia with two spurs. In dorsal view, distinct constriction present between petiole and abdominal segment III (Figure 3B). Abdominal segment IV with tergosternal fusion. Pretergite of abdominal segment IV distinctly differentiated from posttergite; a deep transverse furrow divides them (Figure 6B). Pygostyles present (Figure 4).

Distal margin of abdominal sternum IX concave or convex (Figure 7B). Separation between basimere and harpago usually distinct but indistinct in one species. Basal projection on cuspis well-developed (Figure 9B). Basicoventral portion of aedeagus not extraordinarily expanded. Aedeagus in lateral view, serrate denticles present on basal portion of its ventral margin (Figure 8B).

On forewing (Figure 10B), pterostigma well-developed, radial sector fully present and reaching to costal margin, 2r-rs connected with radial sector posterior to pterostigma, 2rs-m present, cu-a located close to or far from junction between media and cubitus. On hindwing (Figure 11B), radius present or absent, 1rs-m present, media present apical to 1rs-m.

#### Remarks

We resurrect *Stigmatomma* here as an independent genus from a synonymy with *Amblyopone* based on their worker mandible characters (see Discussion of morphological evolution below for detail). No unique male character has been found in *Stigmatomma*. However, males of *Stigmatomma* are distinguished easily from *Adetomyrma* by having a sharper mandible (Figure 12B), constrictions between the petiole and abdominal segment III (Figure 3B) and between AIII and AIV (Figure 6B), serrate denticles on basal margin of the aedeagus (Figure 8B), a complete radial sector and 2rs-m on forewing (Figure 10B), and the 1rs-m on hindwing (Figure 11B); from *Mystrium* by absence of the frontal carina, sharper apex of the mandible, presence of the pygostyle (Figure 4), and less expanded posteroventral portion of the aedeagus; from *Prionopelta* by absence of the frontal carina, more maxillary palpal segments (two in *Prionopelta*, three or four in *Stigmatomma*), two metatibial spurs (one in *Prionopelta*), developed pterostigma, and complete radial sector on forewing; and from *Xymmer* by presence of anterior clypeal conical setae, presence of the pygostyle, having complete radial sector and 2rs-m on forewing, and having 1rs-m on hindwing.

Previous studies most often include diagnostic characters for *Stigmatomma* and/or *Amblyopone* [6], [9]–[12], [17]. Many of these characters are still useful for distinguishing *Stigmatomma* from the other amblyoponine genera, although some taxonomic changes have been made since the information was given. Among the previous diagnostic characters, one should be updated. The number of mesotibial spurs was described as single in Emery [6], but should be updated to one or two, as Bolton mentioned [10]. Brown proposed a new subgeneric classification for *Amblyopone* and discussed the usefulness of wing venation as a diagnostic character of the genus [17]. Brown rejected this classification in 1960 [9] because of an exception in the wing characters observed in *Amblyopone australis*, and proposed a new concept for the genus *Amblyopone* [17]. Yet our results suggest the wing character proposed by Brown [17] is useful to separate among amblyoponine genera (see also the discussion of morphological evolution below).

#### Combination in *Stigmatomma*

The following names are transferred from *Amblyopone* to *Stigmatomma* as **comb. rev.**: *amblyops*, *armigerum*, *bellii*, *bierigi*, *bruni*, *celata*, *chilense*, *denticulatum*, *elongatum*, *emeryi*, *feae*, *impressifrons*, *luzonicum*, *minuta*, *normandi*, *oregonense*, *pallipes*, *quadratum*, *reclinatum*, *rothneyi*, *santschii*, *saundersi*, *silvestrii*, *zwaluwenburgi*.

The following names are transferred from *Amblyopone* to *Stigmatomma* as **comb. nov.**: *agostii*, *annae*, *besucheti*, *boltoni*, *caliginosum*, *cleae*, *crenatum*, *degeneratum*, *egregium*, *electrinum*, *eminia*, *exiguum*, *falcatum*, *ferrugineum*, *fulvidum*, *gaetulicum*, *gingivalis*, *glauerti*, *gnoma*, *gracile*, *groehni*, *heraldoi*, *lucidum*, *lurilabes*, *monrosi*, *mystriops*, *noonadan*, *octodentatum*, *ophthalmicum*, *orizabanum*, *papuanum*, *pertinax*, *pluto*, *punctulatum*, *rubiginoum*, *sakaii*, *smithi*, *trigonignathum*, *trilobum*, *wilsoni*, *zaojun*, and *testaceum* [*testaceum* is a nomen dubium; however, we preliminarily transfer it to *Stigmatomma* based on its locality (Sri Lanka)].

The following species, which are all restricted to Australia, New Caledonia, New Guinea, and New Zealand, remain in *Amblyopone*: *aberrans*, *australis*, *clarki*, *hackeri*, *leae*, *longidens*, *mercovichi*, *michaelseni*.

***Xymmer*** **Santschi, 1914 stat. rev.** (Figures 1E, 2E, 3E, 7E, 8E, 9E, 10E, 11E, 12E, 13E, 14A–C)

*Stigmatomma* (*Xymmer*) Santschi, 1914: 311 [43], original description, type species: *Stigmatomma* (*Xymmer*) *muticum* Santschi, 1914 [43] [lectotype examined (Figure 14A–C). See also Materials and Methods.]

*Stigmatomma* (*Ximmer* [sic]): Emery, 1919 [44].

*Xymmer*: Wheeler, 1922 [7], in key.

*Amblyopone* (*Xymmer*): Clark, 1934 [45].

*Amblyopone* (*Stigmatomma*) (in part): Brown, 1949 [17].

*Amblyopone* (in part): Brown, 1960 [9]; Bolton, 1995 [42]; 2003 [10].

With characters of Amblyoponinae. Frontal carinae absent. Anterior margin of clypeus flat, *without dent-like projections*. Antenna consisting of 13 segments. Mandible with single tooth, apex sharp or blunt (Figure 12E). Palpal formula 3,3 (Figure 13E)/3,2/4,3, one specimen each of six morphospecies dissected. Notaulus distinct (Figure 3E). Mesepimeron with distinct posterodorsal lobe (epimeral lobe: Figure 2E). Mesotibia without spur in most cases, with single spur in one species. Metatibia with two spurs. In dorsal view, distinct constriction present between petiole and abdominal segment III. Abdominal segment IV with tergosternal fusion. Pretergite of abdominal segment IV distinctly differentiated from posttergite; a deep transverse furrow divides them. Pygostyles absent.

Distal margin of abdominal sternum IX convex (Figure 7E). Separation between basimere and harpago distinct. Basal projection on cuspis well-developed (Figure 9E). Basicoventral portion of aedeagus not extraordinarily expanded (Figure 8E). Aedeagus in lateral view, serrate denticles present on basal portion of its ventral margin.

On forewing (Figure 10E), pterostigma well-developed, radial sector absent between M+Rs and 2r-rs, radial sector reaches to costal margin, 2r-rs connected with radial sector posterior to pterostigma, 2rs-m present, cu-a located far from junction between media and cubitus in most cases, located close to junction in one species. On hindwing (Figure 11E), Sc+R1 and radius (R1) absent, 1rs-m absent, media absent apical to 1rs-m.

#### Remarks

We resurrect *Xymmer* here as an independent genus from a synonymy with *Amblyopone* based on a morphological examination of males of nine morphospecies. Males of *Xymmer* can be distinguished from other amblyoponine males in the Malagasy region by a lack of dent-like projections on the anterior margin of the clypeus. In addition to this unique character, *Xymmer* can be separated from *Adetomyrma* by fewer mesotibial spurs (two in *Adetomyrma*, 0–1 in *Xymmer*), absence of the pygostyles, and abdominal sternum IX convex distally; from *Stigmatomma* by lack of the pygostyles, the radial sector on the forewing between Rs+M and 2r-rs, the media on the hindwing apical to the cubitus, and 1rs-m on the hindwing; from *Mystrium* by a lack of the frontal carinae, radial sector on forewing partially absent, an absence of 1rs-m, and media apical to cubitus on hindwing; from *Prionopelta* by a lack of the frontal carinae, having a distinct epimeral lobe, lack of pygostyles, and lack of a vein on hindwing between radial sector and cubitus. The mesotibial spur may be useful as a separable character: the spur is absent in most males of *Xymmer* (only *X*. mgm01 has the spur on the mesotibia out of the nine morphospecies examined), while at least a single spur is present in all males of *Stigmatomma*.

Few morphological discussions exist regarding the taxonomic status of the name *Xymmer*. *Xymmer* was described by Santschi as a monotypic subgeneric taxon under *Stigmatomma* [43]. Since Santschi's original description, *Xymmer* was raised to genus by Wheeler in his identification key for African Amblyoponini [7]. Clark regarded *Xymmer* as a subgenus in *Amblyopone* [45] following Wheeler's suggestion [46]; however, distinguishing characters were not discussed in their treatments. Brown [9], [17] discussed separable characters for *Xymmer* (as a junior synonym under the subgenus *Stigmatomma*) for the first time since Santschi's original description. Brown regarded *Stigmatomma* and its related names as junior synonyms of *Amblyopone* at that time [9], and this treatment constituted the recent concept of the genus *Amblyopone*.

We decided that the male characters proposed above and associations between the worker lectotype and our materials are sufficient to warrant designating *Xymmer* as an independent genus from *Amblyopone*. Santschi's description [43] misidentifies the criterion for separating *Xymmer* from *Amblyopone* as having conical setae just present or absent, as discussed in Brown ([9]: p.165). Although Santschi proposed a lack of conical setae on the anterior clypeal margin in workers as a diagnostic character [43], this description did not provide enough information on the uniqueness of the character observed in *Xymmer*. Our examination of the lectotype of *Stigmatomma* (*Xymmer*) *muticum* found a distinct plate-like projection just dorsal to the junction between the clypeus and labrum, and the projection widens distally with a flat distal margin (Figure 14C). This projection seems to be located along a line slightly ventral to where the conical setae are arranged (Figure 14D), and could replace the function of the conical setae; therefore, it is different from a typical mid-clypeal projection in *Amblyopone*, which bears the conical setae on its anterior margin. This specialized projection in *Xymmer* should be emphasized as a unique character to differentiate *Xymmer* from *Amblyopone* and *Stigmatomma*. The same clypeal character observed in the lectotype of *S*. (*X*.) *muticum* was found in some worker specimens in the Malagasy region, and we confirmed that male specimens identified as *Xymmer* (e.g. CASENT0007085) were congeneric with worker specimens identified as *Xymmer* (e.g. CASENT0007090) (unpublished COI sequence data). The Malagasy workers have a “mutica-like” clypeal character.

#### Combination in *Xymmer*

A single species name, *Xymmer muticus* (Santschi, 1914) comb. rev., is here transferred from the genus *Amblyopone* to *Xymmer*. The genus name *Xymmer* should be treated as masculine in accordance with Article 30.2.4 of the ICZN Code [34], because this name is an anagram for “Myrmex.”

## Discussion

### Morphological evolution of males in the subfamily Amblyoponinae

In this study, we investigated 30 male morphological characters in five amblyoponine genera in the Malagasy region. These characters were sufficient to develop diagnoses for the genera within the Malagasy region. Ten out of 30 characters had genus-specific character states, while three characters had character states that were invariant across all Malagasy amblyoponines. Of the remaining 17 characters, character states are shared among more than one genus. It is of interest to evaluate this pattern of morphological variation in an evolutionary context using trees from recent molecular phylogenetic studies [13]–[15], [36]. One caveat of such an analysis is that our study was focused on species found in the Malagasy region and includes only a limited investigation of amblyoponine genera and species found elsewhere.

To explore character evolution, we chose to map our data on the Brady *et al.* 2006 tree [13], which includes all amblyoponine lineages from Madagascar and the well-supported trees. The Brady *et al.* study [13] did not show strong support for all relationships within Amblyoponinae but did show strong support for two clades of amblyoponines, with one group including *Onychomyrmex*, *Concoctio*, and *Prionopelta* (OCP clade), and the second including *Stigmatomma pallipes* (as *Amblyopone pallipes*), *Adetomyrma*, *Mystrium*, and *Xymmer* (as *Amblyopone mutica*) (XMAS clade). This study did not include *Amblyopone* sensu stricto such as *Am*. *australis*, but did show that *S. pallipes* (as *Am*. *pallipes*) and *X*. *muticus* (as *Am. mutica*) were both within the XMAS clade and do not form a clade exclusive of *Mystrium* and *Adetomyrma*. This study also excluded other Amblyoponine lineages: *Bannapone*, *Myopopone*, *Opamyrma*, and *Paraprionopelta*, which are not known from Madagascar.

We focused on mapping male characters among four genera present in Madagascar: *Adetomyrma*, *Mystrium*, *Stigmatomma*, and *Xymmer* from the XMAS clade; and *Prionopelta* from the OCP clade. *Prionopelta* serves as an outgroup taxon in the analysis of these characters' evolution within the XMAS clade. *Adetomyrma*, *Mystrium*, and *Xymmer* have at least one male character unique to each of the genera (Figure 15). The present study, however, did not find any male character unique to *Stigmatomma* as represented by the species from the Malagasy region. Brown [9], in his generic synopsis of *Amblyopone* (*Amblyopone* sensu lato), also did not indicate any unique characters in his discussion of male characters. In our work, males of *Stigmatomma* are distinguished from the other three taxa in this clade only by a combination of plesiomorphic characters. *Stigmatomma* serves as a practical short-term solution to deal with the balance of taxa placed in *Amblyopone* s. l. (see below). The diversity of characters present in *Stigmatomma* males and workers suggest that further delimitation of this genus will need a careful study as part of a global revision of the Amblyoponinae.

A lack of the pygostyle (character 16) is shared by both *Mystrium* and Malagasy *Xymmer*. Brady *et al.* [13] proposed relationships among four genera in the clade as (*Stigmatomma*+(*Adetomyrma*+(*Mystrium*+*Xymmer*))), though the relationships among them were not well-supported by high posterior probability. Our support of the (*Mystrium*+*Xymmer*) clade by a morphological character is congruent with the molecular results and provides additional evidence for a separation of *Xymmer* from other *Stigmatomma* groups. In all male specimens identified as *Xymmer*, only one specimen from Central African Republic (*X*. cf01: CASENT0087255) has a pygostyle. This exception should be examined in future studies.

No morphological characters have been proposed as synapomorphies for the OCP and XMAS clades. Our morphological study provides a chance to investigate morphological characters that may support the XMAS clade. Our results suggest that the number of teeth on the mandible of the male is a unique character that defines the XMAS clade. The character state single-tooth mandible (character 03) is shared among males in *Adetomyrma*, *Mystrium*, *Stigmatomma*, and *Xymmer*, representing a synapomorphy of the clade. On the other hand, the male of *Prionopelta* possesses a mandible with two teeth.

To determine whether or not this clade-defining character state holds up outside the Malagasy region, we investigated additional genera and *Stigmatomma* species. *Onychomyrmex* males from Australia clearly show a mandible with two teeth, consistent with the molecular finding that *Onychomyrmex* is not part of the XMAS clade. Males of *Myopopone*, a member of the XMAS clade based on Moreau *et al*. 2006 [14], also show the predicted single tooth in specimens observed in this study. Bolton [10] also records *Myopopone* with a single tooth; *Paraprionopelta*, an amblyoponine genus known only from males and not yet included in molecular analysis, shows a single tooth. Thus we predict that *Paraprionopelta* is a member of the XMAS clade, and not related to *Prionopelta* as the name implies. Also missing from molecular analysis are species of *Amblyopone* sensu stricto that includes the type species of the genus *Amblyopone australis*. Brown [[9]: figure 8] illustrated the male mandible of *Amblyopone australis* with two teeth suggesting that *Amblyopone australis* and related species are not members of the XMAS clade and that members of *Stigmatomma* in the XMAS clade (*S. pallipes*) are not congeners with *Am. australis.*

We also predict that males of the genus *Concoctio* have two mandibular teeth, because this genus is nested in the OCP clade with strong support [13], [14]. Though Brady *et al.* [13] had strong support for the placement of *Apomyrma* in Amblyoponinae, its placement within the subfamily is not clear [13]. The mandible of *Apomyrma* males (CASENT0086291, CASENT0086073) appears to be distinctly reduced with no teeth, and thus differs from all other Amblyoponinae species we studied.

### Mandible evolution in *Amblyopone australis* and XMAS clades

This difference in mandibular dentition in males in the OCP and XMAS clades is also reflected in the morphology of their respective worker castes. A comparison of the mandibles of workers of Malagasy *Stigmatomma* and *Amblyopone australis* suggests a possible scenario for worker mandible evolution which involves the parallel evolution of elongate mandibles in the *Stigmatomma* and *Amblyopone* sensu stricto. In *Stigmatomma,* which has single-toothed males, we refer to the worker mandible form as SS (*Stigmatomma* Single) and in *Amblyopone australis*, which has double-toothed males, the worker mandible form is referred to as AD (*Amblyopone* Double).

We propose that the both SS and AD forms developed to hold larger prey, though each acquired this function in a different way as described in the mandible transformations series in Figure 16. It is important to realize that, in both SS and AD forms, the dent-like projections on the basal margin of the mandible (basal denticles) are not on the same plane as teeth on the masticatory margin. The masticatory margin can be further distinguished from the basal margin by the presence of teeth or a ridge along the dorsal side of the mandibular inner face. In some species, however, the angle dividing the basal and masticatory margins is not apparent.

Transformation of worker mandible of Malagasy *Stigmatomma* (SS form with single-toothed mandible in males):

1. Apical teeth curve inwards, such that they remain opposite when mandibles are open (Figure 16-a2).
2. Mandibles become narrower, and basal denticles extend towards apex, below masticatory teeth (Figure 16-a3).
3. Teeth are reduced in size and, at times, form bifurcating teeth when adjacent to basal denticles (Figure 16-a4).
4. The basal-most of the basal denticles (the basal projection) becomes larger (Figure 16-a4).

The basal denticles on the masticatory margin are larger than true masticatory teeth because the basal projections, and not the teeth, are used to hold prey.

Transformation of worker mandible of *Amblyopone australis* (AD form with double-toothed males):

1. Apical teeth do not curve inward and are not opposite when mandibles are open (Figure 16-b2).
2. Mandibles become narrower, the basal denticles do not extend toward apex and remain at base of mandible (Figure 16-b3).
3. Some teeth become larger, never fusing with basal denticles to form bifurcated teeth (Figure 16-b4).

Across the Amblyoponinae, the number of teeth on the male mandible is correlated with the number of major teeth on the masticatory margin of conspecific workers. The male mandible in Amblyoponinae can be interpreted as a “reduced worker mandible” with the following features in comparison with conspecific workers: (a) smaller in length; (b) disappearance of the basal denticles; and (c) disappearance of the masticatory teeth except those which are well-developed on the worker mandible (Figures 16-a4 and a5; b4 and b5). Fusions of adjoining masticatory teeth can occur on the male mandible. On the worker mandible of Malagasy *Stigmatomma* (SS form), only the apical tooth or adjoining apical teeth are well-developed, and all of the others are reduced in size (16-a4). On the other hand, in *Amblyopone australis* (AD form), more than one tooth is distantly developed on the masticatory margin of workers, i.e. the basal tooth and the apical teeth. The difference in the development of masticatory teeth on worker mandibles is reflected in the difference in the number of teeth on the male mandible.

The point on the worker mandible used to hold prey moves distally as this transformation occurs in the AD form. The form of the anterior clypeal margin in workers of *Amblyopone australis* may reflect this change. In workers of *Am. australis*, the anterior margin of the clypeus is somewhat flattened, with smaller conical setae along the margin than in Malagasy species. The SS form mandible might perform better at catching larger prey than the AD form mandible, because the gape of mandibles in the former is wider than in the latter when the basal denticles or teeth on the masticatory margins are opposite each other.

In addition, we found a worker character uniquely observed in Malagasy *Stigmatomma* which distinguishes this genus from the other amblyoponine genera in the Malagasy region: the basal projection on the mandible is distinctly larger than the others arranged on the same margin. This large basal projection was not observed in *Amblyopone australis*. We also confirm that a worker of *Stigmatomma pallipes*, which was used as material in Brady *et al.* [13], has the SS form mandible and the large basal projection. That *S. pallipes* and Malagasy *Stigmatomma* share these mandibular characters suggests they belong in a common clade; the presence of a large basal mandibular projection is likely to be a synapomorphy of this shared group. The large basal mandibular projection seems to function as a stopper when the mandibles are closed.

Worker mandibles similar to the SS form were also observed in workers of *Adetomyrma*, *Mystrium*, and *Xymmer*. In *Adetomyrma*, the length of the mandible is secondarily shortened; in addition, most of the mandibular teeth are combined with the basal denticles on the masticatory margin, and as a result are unrecognizable. However, we confirm that some mandibular teeth remain separated into two layers along dorsal and ventral lines. In *Mystrium*, the mandibles are twisted mesally so that the basal denticles are arranged along the ventral margin of the mandible; the apical tooth is reduced in size and directed ventrally; both the mandibular teeth and the basal denticles are reduced in size and increased in number. Despite this further transformation, all elements of structures typical of the SS form are easily recognizable. In *Xymmer*, both the mandibular teeth and the basal denticles on the masticatory margin are distinct and appear similar to those seen in Malagasy *Stigmatomma*; however, all workers in *Xymmer* lack a large basal projection on the mandible.

*Amblyopone* species sensu Brown [9] with a SS form mandible is separated as a genus rank taxon from the genus *Amblyopone* represented by *Amblyopone australis*, the type species of the genus, based on the following: 1) the SS form mandible is shared among members of the XMAS clade (*Stigmatomma pallipes*, Malagasy *Stigmatomma*, *Adetomyrma*, *Mystrium*, and *Xymmer* workers; 2) SS form mandibles developed independently from AD form mandibles. These findings suggest that *Amblyopone* species with the AD form mandible nest outside the XMAS clade (Figure 15), and that Malagasy *Stigmatomma* belongs in the same clade with *S. pallipes*. Molecular studies support these morphological studies and place *Am*. *australis* as sister to *Prionopelta* (Ward *et al.*, unpubl.; Figure 15). *Stigmatomma* Roger, 1859 is the oldest available name in synonymy under *Amblyopone* and we confirm the SS form mandible and the basal large projection in its type species *Stigmatomma denticulatum* Roger, 1859. Therefore, we resurrect *Stigmatomma* as genus name from synonymy of *Amblyopone* and transfer all species names from *Amblyopone* s. l. with SS form mandibles (including *S. pallipes*+*S. denticulatum*+Malagasy *Stigmatomma*) to *Stigmatomma*.

The mandibular transformations in these two lineages must have occurred after the development of two characters shared between the groups. Amblyoponinae have distinctive anterior clypeal conical setae, which have transformed into a plate-like projection in *Xymmer*. These conical setae are thought to be an apomorphic character of the subfamily Amblyoponinae [10], and are shared in all members of the subfamily. In addition, “the basal denticles” on the basal margin of the mandible are observed not only in Malagasy *Stigmatomma*, *Adetomyrma*, *Mystrium*, and *Xymmer*, but also in *Am*. *australis*, although the denticles in *Am. australis* did not extend to the masticatory margin of the mandible. Both the conical setae and the basal denticles seem to function to hold something large between widely opened mandibles, suggesting that the ancestor might have preyed on animals larger than itself.

### Outside the XMAS clade

Outside the XMAS (*Adetomyrma*+*Mystrium*+*Stigmatomma*+*Xymmer*) clade, the male of *Prionopelta* has character states different from the other four Malagasy amblyoponine genera, some of which appear unique to *Prionopelta*. However, these character states cannot be regarded as unique to the genus, due to the shortage of available morphological data on the males of *Concoctio* and *Onychomyrmex*. *Concoctio* is the sister of *Prionopelta,* and relationships among three genera in the OCP clade ((*Prionopelta*+*Concoctio*)+*Onychomyrmex*) have been strongly supported in Brady *et al.* [13] and Moreau *et al.* [14]. Thus, the discovery of males of *Concoctio* is necessary to evaluate the character states of *Prionopelta*. Using data from Brown's synopsis [9], *Onychomyrmex* shares the same character states with *Prionopelta*: two-segmented maxillary and labial palpi; radial sector on the forewing partially lacking between Rs+M and 2r-rs; hindwing lacking media apical to 1rs-m. These character states are probably shared with *Concoctio* as well. On the other hand, *Onychomyrmex* has different character states from *Prionopelta* in the development of the pterostigma and the location of the 2r-rs radial sector connection on the forewing. These two wing characters, which are seen in *Prionopelta* and are different from those in *Onychomyrmex*, may be generic characters unique to *Prionopelta*.

Advances in molecular ant systematics are creating a new opportunity and demand for morphological studies. Molecular studies will never be able to sequence all species and lineages, and therefore a need to discover morphological characters to diagnose molecular based clades remains. This study demonstrates that males have characters rich and useful for advancing our understanding of ant systematics. For example, the pattern of the number of mandibular teeth in males presents us with a possible scenario for worker mandible evolution that involves the parallel evolution of elongate worker mandibles in Amblyoponinae. We predict that *Paraprionopelta* belongs in the XMAS clade and that *Concoctio* in the OCP clade should have males with two mandibular teeth. These hypotheses can be tested when fresh material is available for sequencing or when males are found for *Concoctio*.
